# Chromatin accessibility uncovers KRAS-driven FOSL2 promoting pancreatic ductal adenocarcinoma progression through up-regulation of CCL28

**DOI:** 10.1038/s41416-023-02313-y

**Published:** 2023-06-28

**Authors:** Shujun Zhang, Peilong Li, Juan Li, Jie Gao, Qiuchen Qi, Guoying Dong, Xiaoyan Liu, Qinlian Jiao, Yunshan Wang, Lutao Du, Hanxiang Zhan, Shuo Xu, Chuanxin Wang

**Affiliations:** 1 Department of Clinical Laboratory, The Second Hospital of Shandong University, 250033 Jinan, Shandong China; 2 Department of Anatomy, School of Basic Medical Sciences, Shandong University, 250012 Jinan, Shandong China; 3 Shandong Institute of Medical Device and Pharmaceutical Packaging Inspection, 15166 Century Avenue, 250101 Jinan, Shandong China; 4 Department of Clinical Laboratory, Shandong Provincial Hospital affiliated to Shandong First Medical University, 250021 Jinan, Shandong China; 5 Department of General Surgery, Qilu Hospital of Shandong University, 250012 Jinan, Shandong China; 6 Department of Neurosurgery, Qilu Hospital of Shandong University, 250012 Jinan, Shandong China

**Keywords:** Pancreatic cancer, Oncogenes, Mechanisms of disease

## Abstract

**Background:**

The epigenetic mechanisms involved in the progression of pancreatic ductal adenocarcinoma (PDAC) remain largely unexplored. This study aimed to identify key transcription factors (TFs) through multiomics sequencing to investigate the molecular mechanisms of TFs that play critical roles in PDAC.

**Methods:**

To characterise the epigenetic landscape of genetically engineered mouse models (GEMMs) of PDAC with or without KRAS and/or TP53 mutations, we employed ATAC-seq, H3K27ac ChIP-seq, and RNA-seq. The effect of Fos-like antigen 2 (FOSL2) on survival was assessed using the Kaplan–Meier method and multivariate Cox regression analysis for PDAC patients. To study the potential targets of FOSL2, we performed Cleavage Under Targets and Tagmentation (CUT&Tag). To explore the functions and underlying mechanisms of FOSL2 in PDAC progression, we employed several assays, including CCK8, transwell migration and invasion, RT-qPCR, Western blotting analysis, IHC, ChIP-qPCR, dual-luciferase reporter, and xenograft models.

**Results:**

Our findings indicated that epigenetic changes played a role in immunosuppressed signalling during PDAC progression. Moreover, we identified FOSL2 as a critical regulator that was up-regulated in PDAC and associated with poor prognosis in patients. FOSL2 promoted cell proliferation, migration, and invasion. Importantly, our research revealed that FOSL2 acted as a downstream target of the KRAS/MAPK pathway and recruited regulatory T (Treg) cells by transcriptionally activating C-C motif chemokine ligand 28 (CCL28). This discovery highlighted the role of an immunosuppressed regulatory axis involving KRAS/MAPK-FOSL2-CCL28-Treg cells in the development of PDAC.

**Conclusion:**

Our study uncovered that KRAS-driven FOSL2 promoted PDAC progression by transcriptionally activating CCL28, revealing an immunosuppressive role for FOSL2 in PDAC.

## Introduction

Pancreatic ductal adenocarcinoma (PDAC) is a devastating human cancer with a 5-year survival rate of less than 10% [1, 2]. Due to its aggressive nature and difficulty in early detection, about 80% of patients with pancreatic cancer are not eligible for surgical resection or receive limited benefits from chemotherapy [3]. Pancreatic cancer lacks effective therapies, and it remains a malignancy associated with a poor prognosis [1]. Therefore, there is an urgent need to further investigate the pathogenesis of PDAC and identify additional therapeutic targets for more effective treatment.

Epigenetic changes, such as chromatin accessibility and histone modifications, play a crucial role in controlling changes in transcriptional programmes [4]. The pattern and identity of open regions on a genome-wide scale provide valuable information on regulatory programmes. Alteration of epigenetic pathways is an emerging mechanism of tumour progression. For example, inactivating mutations of chromatin modifiers have been identified as frequent genetic events in PDAC through whole-genome sequencing studies [5]. Small molecules that target readers, writers, or erasers of histone modifications have shown promising therapeutic effects in mouse models of PDAC [6]. Recent studies have revealed an association between the disruption of large heterochromatin domains and the metastatic transition in PDAC [7]. KRAS mutations are present in over 90% of human pancreatic cancers, serving as an initiating event of PDAC oncogenesis [8, 9]. In contrast, TP53 mutations occur in up to 70% of PDAC cases and are frequently linked to invasive and metastatic phenotypes [10, 11], as supported by genetically engineered mouse models (GEMMs) of PDAC [12]. However, the epigenetic molecular mechanisms through which KRAS and/or TP53 mutants promote PDAC progression remain unknown.

To fill this knowledge gap, we employed GEMMs of PDAC that included wild-type (Pdx-1-Cre), KC (LSL-Kras^G12D/+^; Pdx-1-Cre), and KPC mice (Kras^G12D/+^; LSL-Trp53^R172H/+^; Pdx-1-Cre) to perform assays for transposase-accessible chromatin using sequencing (ATAC-seq), chromatin immunoprecipitation sequencing of H3K27ac (H3K27ac ChIP-seq), and RNA sequencing (RNA-seq). This allowed us to characterise the evolution of the epigenetic landscape and identify specific accessible regions that were associated with tumour immune suppression. By employing clustering and motif analysis, we identified a novel transcription factor (TF) known as Fos-like antigen 2 (FOSL2), which was regulated by mutant KRAS through the MAPK/ERK pathway and enhanced the transcriptional activation of the C-C motif chemokine ligand 28 (CCL28) gene, ultimately promoting the recruitment of regulatory T (Treg) cells. By blocking CCL28 expression upon FOSL2 overexpression, we successfully suppressed Treg cell infiltration and tumour growth, providing insights into the role of FOSL2 in shaping the immune microenvironment of pancreatic cancer.

## Materials and methods

### ATAC-seq, H3K27ac ChIP-seq, and RNA-seq in PDAC mice

For ATAC-seq, H3K27ac ChIP-seq, and RNA-seq analyses, normal pancreas tissues and pancreatic head carcinomas were obtained from WT, KC, and KPC mice at 6 months of age. For KPC mice, primary tumour tissues with liver metastasis, which represented a more aggressive progression, were selected. Each tissue specimen was divided into two parts, with one sent to the pathology department for H&E staining to determine the degree of tumour differentiation and the other frozen at −150 °C. After the pathologists identified the degree of differentiation in the samples, consistent samples with similar degrees of differentiation from WT, KC, and KPC groups were selected for further sequencing separately. For ATAC-seq, frozen samples were processed according to the ATAC-seq protocol by Shanghai Jiayin Biotechnology Ltd. In brief, native nuclei were purified from frozen samples as previously described [13]. The transposition was performed using the Nextera DNA Library Preparation Kit (Illumina), and the transposed DNA fragments were subsequently purified using a MinElute PCR Purification Kit (Qiagen). Samples were amplified by PCR using 1× NEBNext High-Fidelity PCR Master Mix (New England Biolabs, MA). The libraries were purified again with the MinElute PCR Purification Kit (Qiagen) and subjected to sequencing on an Illumina Novaseq 6000 platform using 150-bp paired-end sequencing. ChIP assays were performed by Shanghai Jiayin Biotechnology Co., Ltd. according to the standard crosslinking. The ChIP protocol was performed with modifications. Immunoprecipitation was carried out using an anti-H3K27ac antibody (Abcam, Ab4729, 5 μg), and DNA was extracted and purified using the Universal DNA Purification Kit (#DP214). The ChIP-seq library was prepared using the ChIP-Seq DNA sample preparation kit (NEBNext® Ultra™ II DNA) and sequenced on an Illumina Novaseq 6000 platform using 150-bp paired-end sequencing.

For RNA-seq analysis, libraries were generated using the NEBNext® UltraTM RNA Library Prep Kit for Illumina® (NEB, USA). Briefly, mRNA was purified from total RNA using poly-T oligo-attached magnetic beads. First-strand cDNA was synthesised using M-MuLV Reverse Transcriptase (RNase H-) and random hexamer primers, followed by second-strand cDNA synthesis using DNA Polymerase I and RNase H. The resulting library fragments were purified using the AMPure XP system (Beckman Coulter, Beverly, USA). Size-selected, adaptor-ligated cDNA was treated with USER Enzyme (NEB, USA), followed by PCR amplification using Phusion High-Fidelity DNA polymerase, Universal PCR primers, and Index (X) Primer. The PCR products were then purified again using the AMPure XP system, and library quality was assessed using the Agilent Bioanalyzer 2100 system. Finally, the library preparations were sequenced on an Illumina Novaseq 6000 platform using 150-bp paired-end sequencing.

### ATAC-seq, ChIP-seq, and RNA-seq data analysis

Clean reads from ATAC-seq and ChIP-seq were aligned to the mouse genome (mm10) using Bowtie2 with the parameter “-X 2000” [14]. In ATAC-seq, mitochondrial genome reads were removed from the obtained BAM files. MACS2-callpeak was then used with the parameters “--nomodel -q 0.01 --keep-dup = 1 --SPMR” to call ATAC-seq peaks [15]. Similarly, MACS2-callpeak was used with the parameters “-f BAM -g mm -B -q 0.01 --keep-dup = 1 --SPMR” to call ChIP-seq peaks. Moreover, mm10 blacklist regions were excluded from the called peaks for downstream analysis. The resultant bedGraph files were converted to big wiggle files using the University of California, Santa Cruz (UCSC) bedGraphToBigWig tool [16]. The genomic signal within 2 kb of peaks was visualised using deepTools [17]. Genomic annotations for peaks were computed with Chipseeker [18]. The Binding and Expression Target Analysis (BETA) tool from Cistrome was used to assess the activating or repressive function of selected peaks [19]. HOMER’s findMotifsGenome.pl tool was used to identify enriched TF motifs in the selected peaks [20]. For differential peaks, raw ATAC-seq and ChIP-seq counts for two replicates of all samples were normalised between replicates with size factors computed with DESeq2 [21]. Differentially accessible peaks were filtered based on an adjusted *P* value of <1 × 10^−3^ and a fold-change of at least 3. Differentially expressed H3K27ac regions were filtered based on an adjusted *P* value of <1 × 10^−2^ and a fold-change of at least 2.

RNA-seq data were processed as follows: Clean reads were aligned to the mm10 using the STAR aligner [22]. Gene-level read counts were obtained using FeatureCounts from the Subread package [23]. Raw counts were normalised with DESeq2 [21], and differentially expressed genes (DEGs) were identified using DESeq2 based on an adjusted *P* value of <0.05 and a fold-change of at least 2.

### FOSL2 CUT&Tag

The CUT&Tag assay was performed following previously described protocols with some modifications [24]. Briefly, 10 μL of Concanavalin A-coated magnetic beads (Bangs Laboratories) were added to each sample and incubated at room temperature for 10 min. A 1:50 dilution of anti-FOSL2 (Immunoway, YT1739) or IgG control antibody (Millipore, #12-370) was added and incubated overnight at 4°C. The secondary antibody (Millipore, #AP132) was diluted 1:100 in dig wash buffer, and the cells were incubated at room temperature for 60 min. A 1:100 dilution of the pA-Tn5 adaptor complex was prepared in dig-med buffer (0.01% Digitonin; 20 mM HEPES pH 7.5; 300 mM NaCl; 0.5 mM spermidine; 1 ×  protease inhibitor cocktail) and incubated with cells at room temperature for 1 h. DNA was purified using phenol-chloroform-isoamyl alcohol extraction and ethanol precipitation. A total of 21 μL of DNA was mixed with 2 μL of a universal i5 and a uniquely barcoded i7 primer. A volume of 25 μL of NEBNext HiFi 2× PCR Master mix was added and mixed. The sample was placed in a Thermocycler with a heated lid. Library clean-up was performed using XP beads (Beckman Counter), and sequencing was performed on the Illumina Novaseq 6000 using 150-bp paired-end sequencing.

### FOSL2 CUT&Tag data analysis

Clean reads were mapped to mm10 using Bowtie2 with the parameter “-X 2000” [14]. Peaks were called using MACS2-callpeak with the parameters “-f BAM -g mm -B -q 0.01 --keep-dup=1 --SPMR” [15]. The resulting bedGraph files were converted to bigWiggle files using the UCSC bedGraphToBigWig tool [16]. Genomic signals within 2 kb of peaks were visualised using deepTools [17]. Genomic annotations for peaks were computed with Chipseeker [18]. TF motifs enriched in the selected peaks were identified using HOMER’s findMotifsGenome.pl tool [20].

### Single-cell RNA-seq (scRNA-seq) data mining

We obtained available scRNA-seq data of PDAC samples from the Genome Sequence Archive under project PRJCA001063 and accession number CRA001160 (https://ngdc.cncb.ac.cn/search/?dbId=bioproject&q=PRJCA001063) [25]. All results related to scRNA-seq in this study were obtained from this dataset. We used the Seurat package implemented in R to identify major clusters. Briefly, highly variable genes were generated and used to perform PCA. PCs 1 to 50 were used for clustering to identify distinct groups of cells. These groups were projected onto the t-SNE analysis. We manually characterised the identities of cell types within these groups using well-known PDAC marker genes.

### Mice

Wild-type (Pdx-1-Cre), LSL-Trp53^R172H^, and LSL-Kras^G12D^ mice were obtained from Jackson Laboratory. Double transgenic LSL-Kras^G12D/+^; LSL-Trp53^R172/+^ mice were generated by cross-mating LSL-Trp^53R172H^ and LSL-Kras^G12D^ mice. These mice were further bred with wild-type (Pdx-1-Cre) mice, resulting in different genotypes, including Pdx-1-Cre, LSL-Trp53^R172H^, LSL-Kras^G12D^, KC (LSL-Kras^G12D/+^; Pdx-1-Cre), PC (LSL-Trp53^R172H/+^; Pdx-1-Cre), and KPC (Kras^G12D/+^; LSL-Trp53^R172H/+^; Pdx-1-Cre). Genomic DNA was extracted from mouse tail samples for PCR amplification, and the genotypes of the mice were identified by running nucleic acid gel against the PCR products. We selected wild-type (Pdx-1-Cre), KC (LSL-Kras^G12D/+^; Pdx-1-Cre), and KPC (Kras^G12D/+^; LSL-Trp53^R172H/+^; Pdx-1-Cre) mice for use in the study. Primer sequences for genotyping are listed in Supplementary Table S2. Syngeneic C57BL/6 mice, aged 5–7 weeks, were purchased from Weitonglihua (Peking, China) and housed in specific pathogen-free conditions. All animal procedures and studies were conducted in accordance with the Institutional Animal Care and Use Committee of the Second Hospital of Shandong University.

### Cell culture

MIA PaCa-2 and PANC-1 human PDAC cell lines, PanO2 murine PDAC cell line, and human embryonic kidney 293 T cells were obtained from the Type Culture Collection of the Chinese Academy of Sciences. MIA PaCa-2, PANC-1, and 293 T cells were cultured in high-glucose Dulbecco’s modified Eagle’s medium (Gibco, USA) supplemented with 10% fetal bovine serum (FBS) (Gibco, Carlsbad, CA, USA). PanO2 cells were cultured in Roswell Park Memorial Institute 1640 (RPMI-1640) medium supplemented with 10% FBS. All cell lines were incubated at 37 °C in a humidified atmosphere containing 5% CO_2_.

### Human PDAC tissue microarray (TMA)

The human PDAC TMA, which included 63 tumour tissue specimens and 57 adjacent tissue specimens from 63 patients with PDAC, was obtained from Outdo Biotech, Ltd. (Shanghai, China) (catalogue number: HPan-Ade120Sur-01). The TMA contained detailed clinicopathologic features, including age, gender, grade, TNM stage, and location, as well as prognostic information. FOSL2 antibody was used at a dilution of 1:100. Nuclear staining intensity score was assigned based on the staining intensity (no intensity: 0, weak intensity: 1+, moderate intensity: 2+, and strong intensity: 3+), and the percentage of positive staining was assessed by three experienced pathologists. The final staining index was calculated using the formula: percentage of positive staining × staining intensity score.

### Small interfering RNAs (siRNAs) and stable transfections

siRNAs targeting FOSL2 (siFOSL2#1 and #2) were transfected into 293 T cells using Lipofectamine 2000 (Invitrogen, United States), following the manufacturer’s instructions. Two individual short hairpin RNAs (shRNAs) specific for FOSL2 (shFOSL2 #1 and #2) and KRAS (shKRAS #1 and #2), as well as a negative control shRNA, were synthesised and cloned into the pLent-U6-shRNA plasmid vector (Wzbio, China). The full-length cDNAs of mouse Fosl2 and KRAS^G12D^ were synthesised and cloned into the LV5 plasmid vector (GenePharma, Shanghai, China). All plasmid vectors were extracted using the Endo-Free Plasmid Mini Kit (Omega Bio-Tek, USA). After 24 h, the supernatant of the lentivirus-infected cells was substituted with a complete culture medium. Stable cell lines were established using puromycin (Solarbio, Beijing, China). The sequences of all shRNAs are listed in Supplementary Table S3.

### RNA isolation and real-time PCR

Total RNA was extracted from cultured cells or mouse pancreatic tissues using RNA Fast 2000 Reagent (Fastagen, Shanghai, China) and quantified using a NanoDrop spectrophotometer (Thermo Fisher Scientific, Waltham, MA, USA). The purified RNA was reversely transcribed into cDNA using oligo-dT and random primers with the PrimeScript^TM^ RT reagent Kit (Takara, Dalian, China). Real-time PCR was conducted on a CFX-96 real-time PCR System (Bio-Rad, Shanghai, China) using TB Green^TM^ Premix Ex Taq^TM^ (Takara, Dalian, China). Actin beta (ACTB) was used as the housekeeping gene. The relative expressions of the target genes were calculated using the 2^−ΔΔCT^ method and, subsequently, log2 transformed. Primer sequences are listed in Supplementary Table S2.

### Western blotting analysis

Proteins from PDAC cells or mouse pancreatic tissues were extracted using Western/IP lysis buffer (Beyotime, Haimen, China). Equal amounts of proteins were separated using sodium dodecyl sulfate-polyacrylamide gel electrophoresis (SDS-PAGE) and transferred onto PVDF membranes (Millipore, USA). The membranes were then probed with primary antibodies, followed by incubation with peroxidase-conjugated AffiniPure goat anti-mouse IgG or peroxidase-conjugated AffiniPure goat anti-rabbit IgG. Immunoreactive bands were visualised using the enhanced chemiluminescence system (Bio-Rad Laboratories). ACTB was used as a loading control. The details of all the antibodies used can be found in Supplementary Table S4.

### Cell proliferation assay

Cell proliferation was examined using the Cell Counting Kit-8 (CCK-8) assay (Dojindo Labs). The optical density was assessed with a microplate reader (Bio-Rad, Hercules, CA, United States) at a wavelength of 450 nm.

### Transwell invasion and migration assays

The transwell invasion assay was performed using transwell inserts (Corning, NY, United States) and matrigel (BD Biosciences, San Jose, CA, United States). Briefly, the inserts with 8-µm pores were coated with 8 µL of matrigel. Then, 6 × 10^4^ cells were suspended in 200 µL of serum-free medium and added to the upper compartment of the chamber. Subsequently, 600 µL of RPMI-1640 medium or DMEM containing 20% FBS was added to the lower compartment of the chamber as a chemo-attractant. After incubation for 24 h, cells that invaded through the matrigel were fixed with methanol, stained with 0.1% crystal violet, and photographed using a microscope (Zeiss, Axio Observer). The number of cells in five randomly selected fields from the central and peripheral regions of the filter was counted. The migration assay was conducted in a similar manner without matrigel coating.

### Mouse assay

Male C57BL/6 mice aged 6–8 weeks were subcutaneously injected with 1 × 10^6^ PanO2 cells infected with either vector or oe-FOSL2. Tumour volume was manually measured using slide calipers every 3 days, and mice were monitored throughout the study. On day 21, xenograft tissues were collected upon euthanizing the mice. For antibody treatment, male C57BL/6 mice aged 6–8 weeks were subcutaneously injected with 1 × 10^6^ PanO2 cells infected with either vector or oe-FOSL2. The mice were randomly divided into two groups and were intraperitoneally administered with 3 mg/kg of CCL28 monoclonal antibody (R&D Systems, #MAB533–100) to block CCL28 or isotype IgG once every 3 days. During data collection and analysis, the investigators were blinded to group allocation. All animal experiments were approved by the Institutional Animal Care and Use Committee of the Second Hospital of Shandong University.

### Histopathological analysis

Histopathological analysis of WT, KC, and KPC mice was performed using H&E staining. At least three independent animals were analysed from each group, and five representative, non-overlapping, high-power images were analysed from each slide. One slide was analysed per animal.

### Immunohistochemistry (IHC)

Pancreatic tissues from WT mice and tumour tissues from KC and KPC mice were fixed in 10% formalin for 24 h and then embedded in paraffin. For the IHC test, FFPE sections were deparaffinized in xylene, hydrated using an alcohol gradient, underwent antigen retrieval by water-bath heating, and then blocked with 10% goat serum. The sections were then incubated overnight with primary antibodies at 4 °C. The following day, the corresponding secondary antibody of the primary antibody (HRP labelling) was added to cover the tissue in the ring and incubated at room temperature for 50 min. Visualisation of the antigen localisation was done using the 3,3’-diaminobenzidine (DAB) kit (DAB-0031; Maixin Bio, Fujian, China), and the sections were counterstained with haematoxylin. The primary antibodies for IHC are listed in Supplementary Table S4. At least three independent animals were analysed from each group, and five representative, non-overlapping high-power images were analysed from each slide, with one slide being analysed per animal.

### Immunofluorescence

The paraffin-embedded tissue slices were deparaffinized using xylene and then hydrated with alcohol in a gradient. Antigen retrieval was performed by water-bath heating, followed by overnight incubation with the CD31 primary antibody (GB11063-2, Servicebio) at 4 °C. The secondary antibody was added and allowed to incubate for 1 h at room temperature. Finally, the slices were counterstained with 4′,6-diamidino-2-phenylindole (DAPI). Images were captured using a microscope (Zeiss, Germany) and quantified using ImageJ software.

### FACS analysis

Single cells were incubated with fluorochrome-labelled anti-mouse antibodies as follows: CD45 (#25-0451-82), CD3 (#46-0032-82), CD8a (#17-0081-82), and Foxp3 (#17-5773-82) from eBioscience, and CD4 (#06112-50-100) and CD25 (#07312-60-100) from BioGems. Nuclear proteins were stained using the Foxp3/Transcription Factor Staining Buffer Set (eBioscience). Fluorescence data were acquired using a CytoFLEX Flow Cytometer (Beckman Coulter) and analysed with FlowJo software.

### ChIP assay

ChIP assays were performed using the SimpleChIP Plus Enzymatic Chromatin IP Kit (Magnetic Beads) (CST, #9005). Firstly, chromatin DNA was extracted and sonicated into fragments of 200–400 bp in length. The chromatin fragments were then immune-precipitated with the following antibodies: IgG (CST, #3900) and anti-FOSL2 (CST, #19967). Finally, the precipitated DNA fragments were measured using RT-qPCR. To normalise PCR efficiency, the intensity of the PCR products from the chromatin immunoprecipitates was compared against the intensity of the PCR products of the genomic DNA input, amplified by the same primer pairs. Four primers targeting the upstream region of CCL28 are listed in Supplementary Table S2.

### Dual-luciferase reporter (DLR) gene assay

To perform the DLR assay, pGL3-CCL28 (CCL28-WT) and pGL3-mutated CCL28 (CCL28-mut) plasmids were synthesised and constructed by BoShang (BoShang, China). 293 T, MIA PaCa-2, and PANC-1 cells were co-transfected with the luciferase reporter plasmid and siRNA or shRNA of FOSL2. The luciferase assays were performed 48 h after transfection using the Dual-Luciferase Reporter Assay System kit (Promega, USA). The relative luciferase activity was then measured and normalised to Renilla activity.

### Enzyme-linked immunosorbent assay (ELISA)

The concentrations of CCL28 and VEGFA in the culture supernatant of human PDAC cell lines or mouse serum were measured using the CCL28 ELISA kit (human, DY717; mouse, DY533) or VEGFA ELISA kit (mouse, DY493) following the manufacturer’s instructions (R&D Systems). The protein concentration of cellular supernatant or serum was quantified using the BCA Protein Assay Reagent (Thermo Scientific Scientific).

### Statistical analysis

Statistical analysis was performed using R software (version 3.6.0), SPSS 17.0 (IBM, SPSS, Chicago, IL, United States), and GraphPad Prism 5.0 (GraphPad Software, La Jolla, CA, United States). The Chi-square test was used to assess the statistical significance of the association between FOSL2 protein levels and clinicopathologic characteristics. Survival curves were estimated using the Kaplan–Meier method and compared using log-rank tests. The potential risk factors of FOSL2 on survival time were assessed using multivariate Cox regression analysis. The correlation between FOSL2 and CCL28 protein levels was calculated using Pearson correlation analysis. Student’s *t* test was used for comparisons between two groups, while ANOVA *F*-test was used for comparisons among three groups. A *P* value <0.05 was considered statistically significant.

## Results

### Dynamic changes of accessible chromatin in PDAC mouse models

**Fig. 1:**
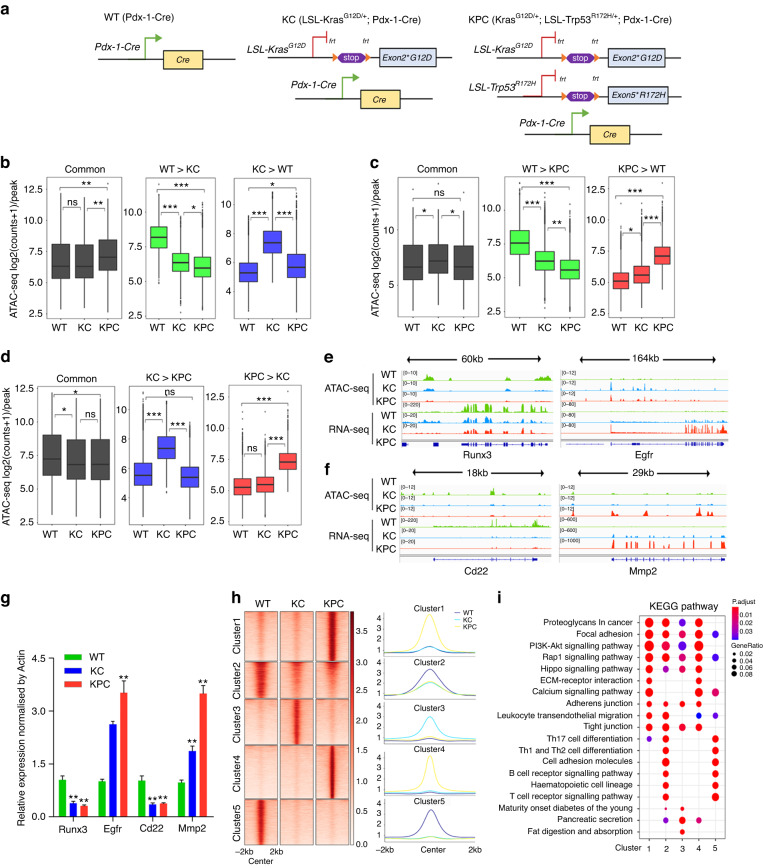
Dynamic changes of accessible chromatin in PDAC mouse models. **a** Genetic strategy for KC (LSL-Kras^G12D/+^; Pdx-1-Cre) and KPC (Kras^G12D/+^; LSL-Trp53^R172H/+^; Pdx-1-Cre) genetically engineered mice. **b**–**d** Boxplots of ATAC-seq counts per peak from the indicated samples (labelled at the bottom) at common or differentially accessible regions from the comparison labelled above. Box indicates interquartile range with whiskers ±1.5 times this range and outlier points. *P < 0.05; **P < 0.01; ***P < 0.001, n.s. not significant. **e**, **f** ATAC-seq and RNA-seq tracks around Runx3, Egfr, Cd22, and Mmp2 in WT, KC, and KPC mice. **g** RT-qPCR assay was performed to detect the expression levels of indicated genes (labelled at the bottom) in WT, KC, and KPC mice. *n* = 4 for each group. The KC and KPC mice were compared with WT, respectively. *P < 0.05; **P < 0.01. **h** Heatmaps (left panel) and average intensity curves (right panel) of k-means clustered ATAC-seq signal for all differentially accessible regions identified from comparisons between WT, KC, and KPC mice. **i** Dotplot showing the representative KEGG pathway terms enriched in each cluster based on the adjusted p-value. The pathways were ranked and displayed based on an ascending adjusted *P* value.

We employed KC (LSL-Kras^G12D/+^; Pdx-1-Cre) and KPC (Kras^G12D/+^; LSL-Trp53^R172H/+^; Pdx-1-Cre) mice to investigate the epigenetic changes of pancreatic cancer (Fig. 1a). Histological analysis revealed that WT mice displayed normal pancreatic morphology, KC mice showed low-grade pancreatic intraepithelial neoplasia (PanIN) lesions, and in contrast, KPC mice developed diffuse ductal invasive adenocarcinoma (Fig. S1A). IHC analysis demonstrated that the levels of the cell proliferation marker Ki-67 progressively increased in KPC and KC mice compared to WT mice (Fig. S1B). We performed ATAC-seq, H3K27ac ChIP-seq, and RNA-seq on WT, KC, and KPC mice (*n* = 2 per group) to explore the dynamic epigenetic landscape (Supplementary Table S1). Principal component analysis (PCA) indicated that the biological replicates clustered together within the same group but were clearly separated between different groups (Fig. S1C–E). The accessible regions were frequently found in promoters, introns, and distal intergenic regions (Fig. S1F).

We identified a total of 79,598 ATAC-seq regions, out of which more than 13,000 were found to be differentially regulated when comparing KC and WT mice (Fig. S1G). Approximately 17,000 were differentially regulated between KPC and KC mice (Fig. S1I), while KPC and WT mice exhibited the largest number of differentially accessible regions, with approximately 24,000 (Fig. S1H). We observed that regions exhibiting less or more accessibility in KC mice compared to WT mice tended to display less or more accessibility in KPC mice as well (Fig. 1b). For example, the tumour suppressor gene Runx3 [26] displayed decreased accessibility in both KC and KPC mice compared to WT mice (Fig. 1e), while the oncogene Egfr [27] exhibited higher signal in KC and KPC mice compared to WT mice (Fig. 1e). Similarly, regions displaying lower or higher signal in KPC mice compared to WT mice also showed lower or higher signal in KC mice compared to WT mice (Fig. 1c), as exemplified by the Cd22 and Mmp2 loci, respectively (Fig. 1f). Previous studies have shown that Cd22 can suppress tumourigenesis, while Mmp2 can promote tumourigenesis [28, 29]. Our RNA-seq data also showed the same trend when comparing KC vs. WT and KPC vs. WT (Fig. S1J, K). In the comparison of KPC vs. KC (Fig. 1d), the open accessibility in KPC mice relative to KC mice showed a higher signal than WT mice, which was consistent with the RNA-seq data showing higher expression levels in KPC compared to WT mice (Fig. S1L). Taken together, these comparisons identified regions and expressed genes that were stably altered in WT mice after KRAS or/and TP53 mutations. To better understand the chromatin-transcription relationships, we used BETA [19] to integrate the gene expression changes with chromatin accessibility signals for pairwise comparisons of WT, KC, and KPC mice, respectively. Our results showed that the gained peaks were highly correlated with up-regulated genes but not down-regulated genes (Fig. S2A), while the lost peaks were highly related with down-regulated genes rather than up-regulated genes in each comparison (Fig. S2B). For example, the expression levels of the illustrated four genes were positively correlated with corresponding chromatin accessibility (Fig. 1e, f). We validated the expressions of the four genes in samples of the PDAC mouse model using RT-qPCR (Fig. 1g).

To identify accessible regions that were biased toward WT, KC, or KPC mice, we intersected the differentially accessible regions from each comparison. When compared to “not” regions (Fig. S2D), more regions were specific to WT, KC, and KPC mice (Fig. S2C). For RNA-seq, there were more regions specific to or absent in WT mice than in KC and KPC mice (Fig. S2E, F). We performed gene ontology (GO) analysis using the compareCluster function of the R package clusterProfiler for these specific or absent peaks. We identified that some immune-associated pathways, such as lymphocyte proliferation, differentiation, and T-cell activation, were absent in KC and KPC mice (Fig. S2G). In line with this result, RNA-seq also showed that immunologically relevant pathways were lost in KC and KPC mice (Fig. S2H).

We identified 35,452 regions that were differentially accessible in at least one pairwise comparison of WT, KC, and KPC mice. Using k-means clustering, we partitioned all 35,452 differentially accessible regions into five clusters. Clusters 2 and 5 were enriched in WT, cluster 3 was enriched in KC, and clusters 1 and 4 favoured KPC (Fig. 1h). We compared the expressions of genes within 20 kb of peaks in each cluster. We found that clusters 1, 3, and 4 were positively correlated with significant changes in RNA expression compared to WT (Fig. S2I), and cluster 5 showed higher RNA expression in WT than in KC and KPC, indicating that chromatin accessibility was involved in the epigenetic regulation of gene expression. However, cluster 2 showed no difference in RNA expression among the three groups (Fig. S2I), implying the presence of other possible regulatory mechanisms on gene expression. Kyoto Encyclopedia of Genes and Genomes (KEGG) analysis for each cluster showed that clusters 1 and 4 favoured KPC and were enriched in the ECM-receptor interaction and calcium signalling pathways but were absent in immune-associated signalling compared to clusters 2 and 5 (Fig. 1i). These results indicated that changes in chromatin accessibility might promote PDAC tumourigenesis and progression by inhibiting immune-related signalling.

### Differential H3K27ac profiling in PDAC mice

**Fig. 2:**
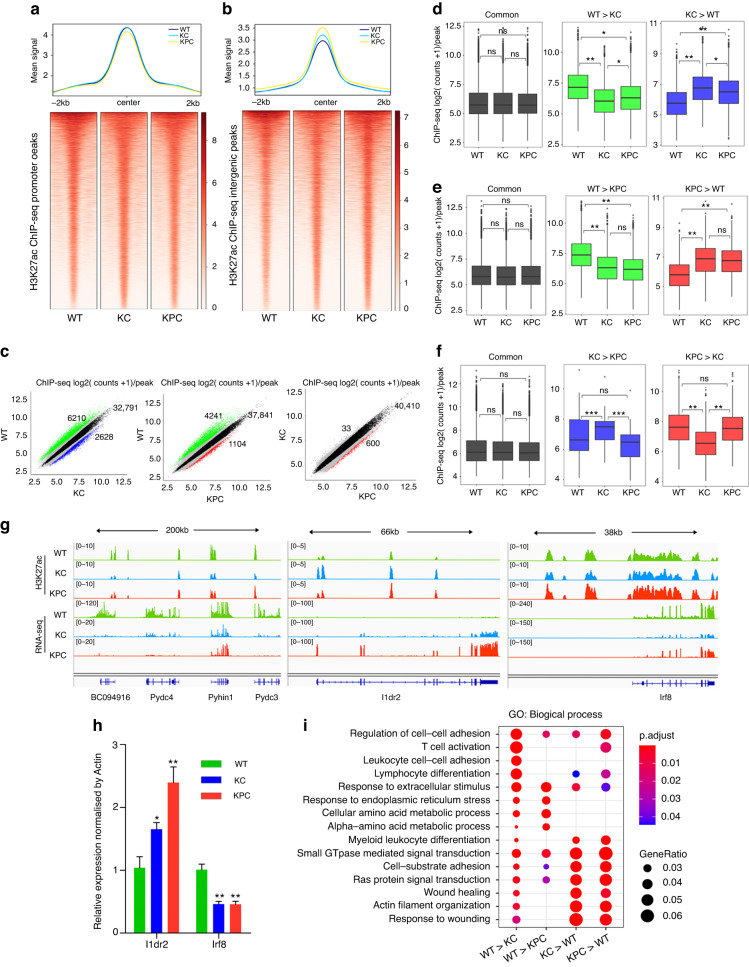
Changes of H3K27ac signals in the PDAC mouse model. **a** The average intensity curves (top panel) and heatmaps (bottom panel) for H3K27ac signals at promoter regions centred on the summit of peaks in WT, KC, and KPC mice. The region plotted comprises +/−2 Kb around the summit. **b** The average intensity curves (top panel) and heatmaps (bottom panel) for H3K27ac signals at intergenic regions centred on the summit of peaks in WT, KC, and KPC mice. The region plotted comprises +/−2 Kb around the summit. **c** Scatterplots of mean H3K27ac counts per peak comparing the indicated samples. Red indicates differential peaks enriched in KPC compared with WT or KC; Blue indicates differential peaks enriched in KC compared with WT or KPC; Green indicates differential peaks enriched in WT compared with KC or KPC; Black indicates common peaks whose fold-change is less than 2; Grey indicates the rest of peaks whose fold-change is greater than 2 as well as adjusted p-value greater than 1 × 10^-2^. The upper-right number showed the count of common peaks. **d**–**f** Boxplots of H3K27ac counts per peak from the indicated samples (labelled at the bottom) at common or differentially H3K27ac regions from the comparison labelled above. Box indicates interquartile range with whiskers ±1.5 times this range and outlier points. **g** H3K27ac ChIP-seq and RNA-seq tracks around Pydc3, Ildr2, Irf8, etc., in WT, KC, and KPC mice. **h** RT-qPCR assay was performed to detect the expressions of indicated genes (labelled at the bottom) in WT, KC, and KPC mice. *n* = 4 for each group. The KC and KPC mice were compared with WT, respectively. *P < 0.05; **P < 0.01. **i** GO term analysis of differential H3K27ac peaks in indicated groups (labelled at the bottom).

We identified a total of 65,485 H3K27ac peaks, with signals frequently observed in promoters, introns, and distal intergenic regions (Fig. S2J). The global signals at promoters showed no difference among the three groups (Fig. 2a), but the signals at intergenic regions were progressively higher in KPC and KC mice compared to WT mice (Fig. 2b). Approximately 8800 and 5300 H3K27ac peaks were differentially expressed in KC vs. WT and KPC vs. WT, respectively, but only 633 differential H3K27ac peaks were identified in KPC vs. KC (Fig. 2c), indicating minimal differences in H3K27ac signals between KPC and KC mice. Regions with lower or higher H3K27ac signals in KC than WT mice also tended to have lower or higher signals in KPC mice (Fig. 2d). A similar pattern was observed in the comparison between KPC and WT mice (Fig. 2e). For instance, neighbouring genes of BC094916, Pydc4, Pyhin1, and Pydc3 loci had lower H3K27ac signals in KC and KPC mice than in WT mice (Fig. 2g), while gene Ildr2 had higher signals in KC and KPC mice than in WT mice (Fig. 2g). Despite the fewer number of differential H3K27ac peaks between KPC and KC mice (Fig. 2c), most of these peaks were KC-specific (Fig. 2f), as exemplified by the Irf8 locus, where H3K27ac signals were increased in KPC and WT mice compared to KC mice at its promoter and downstream region (Fig. 2g). The RNA-seq expressions of the Ildr2 and Irf8 genes also showed a similar trend with H3K27ac signals (Fig. 2g). Furthermore, we validated the expressions of these two genes in PDAC mouse samples by RT-qPCR (Fig. 2h). In line with the GO results of our ATAC-seq and RNA-seq, immune-associated pathways, such as T cell activation, leucocyte cell–cell adhesion, and lymphocyte differentiation, were suppressed or absent in KC and KPC mice (Fig. 2i), implying that the suppression of immune signalling played an important role in PDAC progression.

### Motif analyses of differentially accessible regions reveal novel regulators

**Fig. 3:**
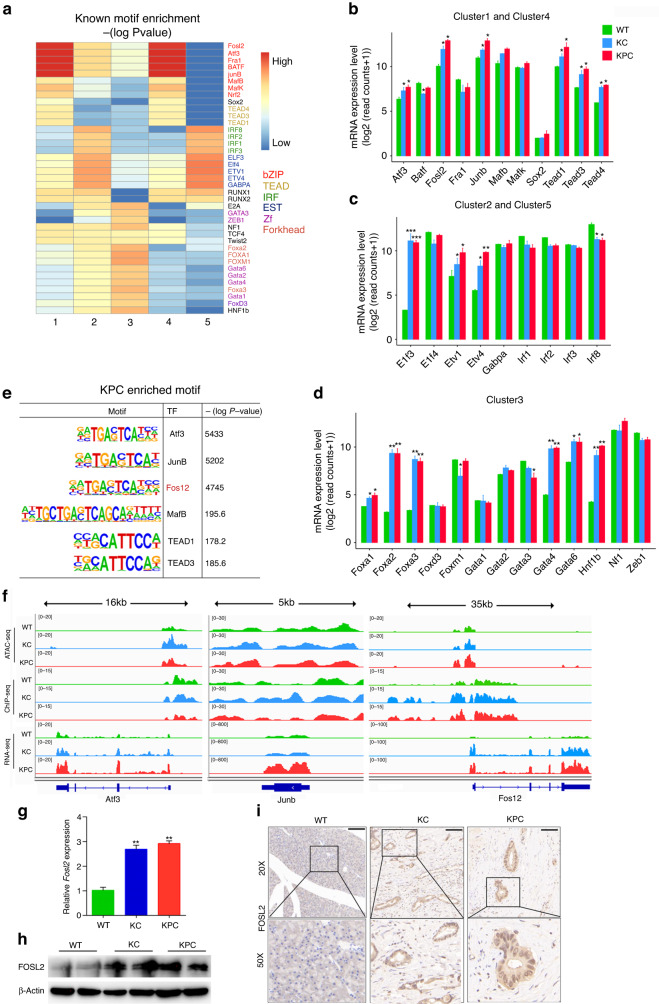
TF FOSL2 expression is up-regulated in PDAC mouse models. **a** Enrichment of all known motifs within each cluster of accessible regions. All motifs with an enrichment −(log *P* value) with a base of 10. TF categories are indicated on the right side. The motifs upon the −(log *P* value) with a base of 10 of motifs greater than 150 for clusters 1–5, separately, were shown. **b**–**d** Analysis of mRNA levels of TFs associated with clusters 1–5 in WT, KC, and KPC mice. The KC and KPC mice were compared with WT, respectively. *P < 0.05; **P < 0.01; ***P < 0.001. **e** Representative KPC-specific motifs and corresponding candidate TFs. **f** Genome browser view of ATAC-seq, H3K27ac ChIP-seq, and RNA-seq coverage at the Atf3, Junb, and Fosl2 loci in WT, KC, and KPC mice. **g** RT-qPCR assay was performed to detect the Fosl2 expression in WT, KC, and KPC mice. *n* = 4 for each group. The KC and KPC mice were compared with WT, respectively. **P < 0.01. **h** Immunoblot showing the abundance of FOSL2 in WT, KC, and KPC mice. **i** Representative images of IHC staining for FOSL2 in WT pancreas, KC, and KPC neoplastic tissues. Scale bar, 100 μm. ***P < 0.01.

*Cis*-regulatory elements play a significant role in oncogenesis and progression by typically binding with TFs [30]. We identified enriched motifs and corresponding TFs in clusters 1–5, with clusters 1 and 4 enriched for bZIP and TEAD motifs, clusters 2 and 5 enriched for IRF and EST families, and cluster 3 enriched for Forkhead and Zf TFs (Fig. 3a). Some TFs, such as NRF2 and FOXA1 [31, 32], have been shown to be important in PDAC progression. RNA-seq data demonstrated that most of the TFs enriched in cluster 3 were more differentially expressed in KC and KPC compared to WT than those in other clusters (Fig. 3b–d). We then focused on the TFs enriched in KPC and with higher expression in KPC and KC than in WT mice (Fig. 3e). Our findings showed that Fosl2 had enriched open chromatin, H3K27ac signal, and higher RNA expression in KC and KPC than in WT (Fig. 3f). However, the ATAC-seq and ChIP-seq signal of Atf3 and JunB loci did not show consistency with RNA expression data (Fig. 3f). Although there have been reports on the function and mechanism of Atf3 and JunB in PDAC [33, 34], the underlying mechanism of FOSL2 in PDAC remains unknown. We confirmed the higher expression of FOSL2 in KPC and KC compared to WT mice by RT-qPCR, Western blotting, and IHC (Fig. 3g–i). Therefore, we focused on FOSL2 for further study.

### FOSL2 expression is up-regulated and associated with poor prognosis of patients with PDAC

**Fig. 4:**
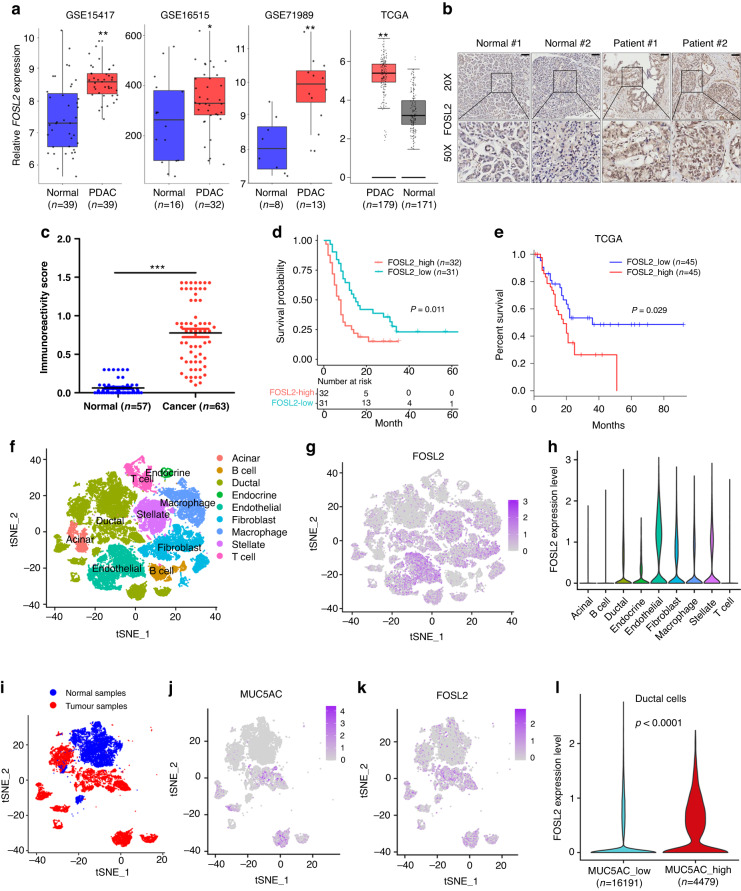
FOSL2 expression is up-regulated and associated with poor prognosis in clinical PDAC samples. **a** Boxplots showing the expression levels of FOSL2 in PDAC patients from GSE15417, GSE16515, GSE71989, and TCGA datasets. The types of GSE data are all gene expression microarrays on human PDAC. *P < 0.05; **P < 0.01. **b** Representative images of IHC staining for FOSL2 in adjacent (*n* = 57) and carcinoma (*n* = 63) tissues of PDAC samples. **c** Quantification of IHC staining for FOSL2 in adjacent (*n* = 57) and carcinoma (*n* = 63) tissues of PDAC samples. ***P < 0.001. **d** OS of PDAC patients grouped by the IHC scores of FOSL2. **e** OS of PDAC patients grouped by FOSL2 mRNA expression. The survival data were obtained from the TCGA database. The high and low expression groups were defined using the quartile as the group cutoff. **f** The *t*-distributed stochastic neighbour embedding (t-SNE) plot demonstrates the main cell types in PDAC. **g** Expression levels of FOSL2 for main cell types are plotted onto the t-SNE map. The colour key from grey to purple indicates relative expression levels from low to high. The “expression level” was normalised by the logNormalize method in Seurat. **h** Violin plots displaying the expression of FOSL2 across the cell types identified in PDAC. The y-axis shows the normalised read count. **i** t-SNE plot demonstrates the distribution of carcinoma and adjacent samples in PDAC patients. Blue indicates normal samples, and red indicates tumour samples. **j**, **k** Expression levels of MUC5AC and FOSL2 genes in a ductal cell are plotted onto the t-SNE map. The colour key from grey to purple indicates relative expression levels from low to high. **l** Violin plot showing the global FOSL2 expression level from scRNA-seq in the low MUC5AC ductal cell group and high MUC5AC ductal cell group.

To investigate the expression of FOSL2 in human samples, we initially used data from three GEO datasets and TCGA, which revealed that FOSL2 mRNA was highly expressed in PDAC samples compared to normal samples (Fig. 4a). Next, we performed IHC analysis to examine the expression of FOSL2 at the protein level in a TMA consisting of 63 PDAC samples and 57 matched adjacent normal tissues. The results showed that the expression of FOSL2 was significantly up-regulated in PDAC tissues compared to non-tumour tissues (Fig. 4b, c). Moreover, high FOSL2 expression was positively correlated with poor prognosis (Fig. 4d), which was consistent with the TCGA RNA-seq data showing that the high FOSL2 group was associated with poor outcomes in PDAC patients (Fig. 4e). Univariate and multivariate Cox proportional hazards regression analyses demonstrated that FOSL2 was an independent prognostic factor for overall survival (OS) of PDAC (Table 1). We also explored the relationship between FOSL2 expression and clinical pathological factors in 63 PDAC patients and found that higher FOSL2 expression was positively correlated with high tumour grade (*P* = 0.044) but had no association with other clinical characteristics (Supplementary Table S5).

**Table 1:** Univariate and multivariable Cox regression analysis with covariates including age, gender, tumour location, grade, TNM stage, and FOSL2 IHC score for overall survival.

Factor	Univariate					Multivariate				
	Coef	HR	Se	*z*	*p*	Coef	HR	Se	*z*	*p*
Age: ≥67 vs <67	0.372	1.451	0.285	1.307	0.191					
Gender: M vs F	0.191	1.211	0.288	0.664	0.506					
Location: head vs tail	0.46	1.585	0.3	1.536	0.124					
Grade: III vs I–II	0.559	1.749	0.285	1.959	**0.05**	0.407	1.502	0.3	1.355	0.175
T: T3 vs T1–T2	0.302	1.353	0.422	0.716	0.474					
N: Yes vs No	0.408	1.503	0.3	1.36	0.174					
M: M1 vs M0	1.284	3.611	0.543	2.364	**0.018**	0.804	2.234	0.568	1.416	0.157
FOSL2: high vs low	0.734	2.083	0.29	2.529	**0.011**	0.632	1.882	0.299	2.112	**0.035**

Bold values indicate statistical significance.

*Coef* regression coefficient, *HR* hazard ratio, *Se* standard error of coef.

To explore the specific cell type of FOSL2 expression, we reanalysed public scRNA-seq data consisting of 24 PDAC tumour tissues and 11 control pancreas tissues. We conducted clustering and cell type annotation analysis (Figs. S3A and 4f) and displayed well-known markers across the cell types identified in PDAC to demonstrate the accuracy of cell type annotation (Fig. S3B). Our findings revealed that FOSL2 expression was very low in T, B, and acinar cells but enriched in endothelial, ductal, and other types of cells (Fig. 4g, h). We focused on the expression of FOSL2 within pancreatic ductal cells. Previous studies have reported that MUC5AC is only expressed in 2–4% of the healthy pancreas [35], while approximately 75–92% of PDAC cases show expression of this marker [36, 37]. Our T-SNE plot showed that MUC5AC was represented in ductal cells but absent in other types of cells (Fig. S3C). We observed that the expression of MUC5AC was absent in ductal cells from normal samples but enriched in part ductal cells from tumour samples (Fig. 4i, j), while FOSL2 was up-regulated in ductal cells from tumour samples compared to ductal cells from normal samples (Fig. 4i, k). We also detected a positive relationship between MUC5AC and FOSL2 expressions in PDAC ductal cells (Fig. 4j, k). We divided these ductal cells into two groups based on their MUC5AC expression levels. The high MUC5AC group was defined by raw expressed counts of MUC5AC greater than zero, while the remaining cells were classified as the low MUC5AC group (Fig. S3D). We found that the high MUC5AC group showed higher FOSL2 expression compared to the low MUC5AC group (Fig. 4l). These results suggested that FOSL2 was up-regulated in cancerous ductal cells compared to normal pancreatic ductal cells and could serve as a novel prognostic biomarker in patients with PDAC.

### FOSL2 promotes PDAC progression and immune evasion

**Fig. 5:**
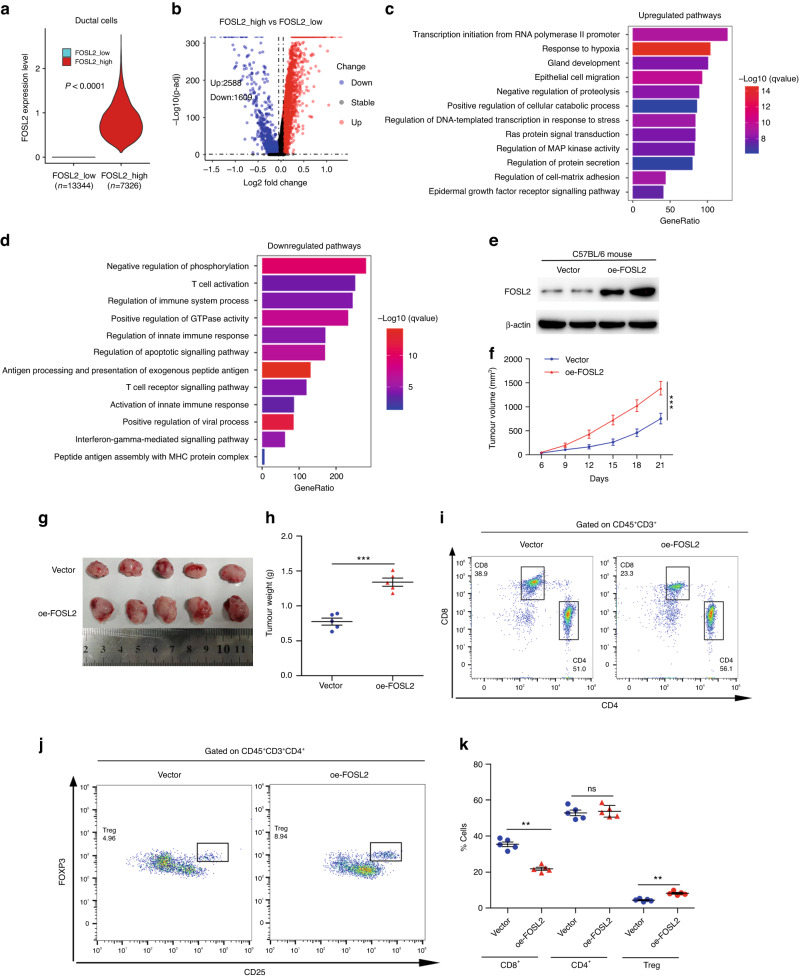
FOSL2 promotes PDAC immunosuppression. **a** Violin plot showing the global FOSL2 expression level from scRNA-seq in the low FOSL2 ductal cell group and high FOSL2 group. **b** Volcano plot exhibited differentially expressed RNA in the high FOSL2 group vs. the low FOSL2 group. **c** GO term analysis showed the up-regulated signalling pathway in the high FOSL2 group compared with the low FOSL2 group. **d** GO term analysis showed the down-regulated signalling pathway in the high FOSL2 group compared with the low FOSL2 group. **e** Immunoblot showing the abundance of FOSL2 in tumour xenograft of indicated groups. **f** PanO2 cells transfected with Vector or oe-FOSL2 were injected subcutaneously into C57BL/6 mice. Tumour volume was monitored and measured. Data are shown as mean ± SD. ***P < 0.001. **g** Representative images show tumour volume in the indicated groups. **h** Graphical quantification of difference in tumour weight on day 21 in Vector or oe-FOSL2 cohorts. Data are shown as mean ± SEM. ***P < 0.001. **i** Representative flow staining of CD4^+^ and CD8^+^ T cells gated on CD45^+^CD3^+^ cells in tumour xenograft of indicated groups. **j** Representative flow staining of CD25^+^Foxp3^+^ Treg cells gated on CD45^+^CD3^+^ CD4^+^ cells in tumour xenograft of indicated groups. **k** The frequency of CD8^+^, CD4^+^, and Treg cells in tumour xenograft of the indicated groups. **P < 0.01; n.s. not significant.

We divided ductal cells from scRNA-seq into two groups based on FOSL2 expression. The high FOSL2 group was defined as having raw FOSL2 counts greater than zero, while the rest of the ductal cells were classified as the low FOSL2 group (Fig. 5a). We observed 2,588 up-regulated and 1,609 down-regulated genes in the high FOSL2 group compared to the low FOSL2 group (Fig. 5b). GO enrichment analysis of these up-regulated or down-regulated genes revealed activation of pathways involved in Ras signal transduction, epithelial cell migration, and MAP kinase activity (Fig. 5c). In contrast, we observed suppression of immune-associated pathways, such as T-cell activation, immune system process, and innate immune response in the high FOSL2 group (Fig. 5d). We also experimentally investigated the biological functions of FOSL2 both in vitro and in vivo. The depletion or overexpression of FOSL2 was confirmed by RT-qPCR and Western blotting analysis (Fig. S4A–C). Results from the CCK-8 assay indicated that FOSL2 knockdown reduced cell proliferation compared to the control group in MIA PaCa-2 and PANC-1 cells (Fig. S4D, E). In contrast, overexpression of FOSL2 promoted cell proliferation in MIA PaCa-2, PANC-1, and PanO2 cells (Fig. S4J–L). Furthermore, the transwell assay showed that FOSL2 depletion impaired migration and invasion (Fig. S4F–I), while overexpression promoted cell migration and invasion (Fig. S4M–O).

We found that immune signalling was suppressed in the high FOSL2 group (Fig. 5d), implying that FOSL2 might promote PDAC progression by inhibiting the tumour immune microenvironment. To investigate this hypothesis, we injected murine PanO2 cells transfected with vector or oe-FOSL2 lentivirus subcutaneously into syngeneic C57BL/6 mice (Fig. 5e). Strikingly, FOSL2 overexpression significantly accelerated tumour growth (Fig. 5f–h). Immune cell composition analysis revealed a notable reduction in CD8^+^ T cell infiltration in oe-FOSL2 tumours (Fig. 5i, k). Although the percentages of CD4^+^ cells were similar in oe-FOSL2 and vector tumour samples (Fig. 5i, k), the number of CD4^+^CD25^+^ Foxp3^+^ Tregs was increased in oe-FOSL2 tumours (Fig. 5j, k). In summary, these results indicated that FOSL2 might drive PDAC progression and immune evasion.

### FOSL2 transcriptionally activates CCL28 by binding to CCL28 upstream

**Fig. 6:**
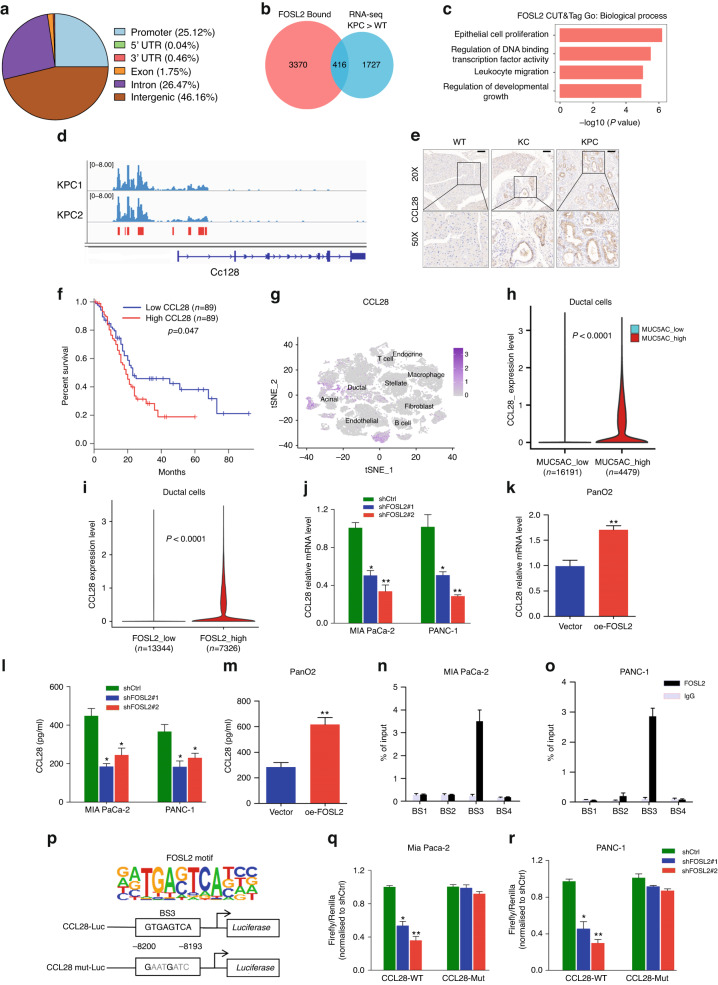
CCL28 is a transcriptional target of FOSL2. **a** Genomic distribution of FOSL2 CUT&Tag peaks. 5′UTR, 5′ untranslated region. 3′UTR, 3′ untranslated region. **b** Venn diagram outlining the overlap between FOSL2 target gene associations and 2,143 highly expressed genes in the KPC transcriptome than WT. **c** GO term analysis of FOSL2-regulated genes. **d** CUT&Tag signal track showing Ccl28-associated FOSL2 occupancy in KPC mice. **e** Representative images of IHC staining for CCL28 in WT pancreas, KC, and KPC neoplastic tissues. Scale bar, 100 μm. **f** OS of PDAC patients grouped by CCL28 mRNA expression. The survival data were obtained from the TCGA database. The high and low expression groups were defined using the median as the group cutoff. **g** Expression level of CCL28 for main cell types are plotted onto the t-SNE map. The colour key from grey to purple indicates relative expression levels from low to high. The “expression level” was normalised by the logNormalize method in Seurat. **h** Violin plot showing the global CCL28 expression level from scRNA-seq in the low MUC5AC ductal cell group and high MUC5AC ductal cell group. **i** RNA expression level of CCL28 in the indicated group in PDAC ductal cells from scRNA-seq data. The statistical test was carried out using the Wilcoxon rank-sum test. **j**, **k** CCL28 protein levels in MIA PaCa-2 and PANC-1 pancreatic cancer cells with FOSL2 knockdown (**j**) or in PanO2 cells with FOSL2 overexpression (**k**). For (**j**), the shFOSL2#1 and shFOSL2#2 groups were compared with the shCtrl group, respectively. **l**, **m** CCL28 protein in supernatants from MIA PaCa-2 and PANC-1 cells with FOSL2 knockdown (**l**), or from PanO2 cells with FOSL2 overexpression (**m**), as determined by ELISA. For (**l**), the shFOSL2#1 and shFOSL2#2 groups were compared with the shCtrl group, respectively. **n**, **o** ChIP analysis of the binding of FOSL2 to the CCL28 upstream in MIA PaCa-2 (N) and PANC-1(O) cells. **p** FOSL2 motif (top) and scheme of CCL28 upstream luciferase reporter constructs illustrating the wild-type or mutated sequences of potential FOSL2 binding sites (bottom). **q**, **r** Luciferase reporter activities of CCL28 upstream or upstream with mutated binding sites in MIA PaCa-2 (**q**) and PANC-1 (**r**) cells with stable FOSL2 knockdown. The shFOSL2#1 and shFOSL2#2 groups were compared with the shCtrl group, respectively. Data are representative of at least three independent experiments and shown as mean ± SEM. *P < 0.05; **P < 0.01.

The genome-wide occupancy and direct target genes of FOSL2 in PDAC have not been studied. Therefore, we performed CUT&Tag with FOSL2 antibody from two KPC mice to identify its binding sites on a genome-wide scale [24, 38]. In total, we identified 7,812 FOSL2 peaks, and the global FOSL2 signals between the two replicates were similar (r = 0.90) (Fig. S5A, B). These peaks were mainly localised to promoters, introns, and distal intergenic regions (Fig. 6a), suggesting that FOSL2-directed regulation occurred at promoters and enhancers. Co-TF motif analysis identified Six1, C/EBPβ, PRDM14, Elk1, and AP-2α TFs, which are known to be correlated with PDAC progression (Fig. S5C) [39–43]. This finding suggested their transcriptional cooperation with FOSL2. We intersected the FOSL2 binding regions with the up-regulated genes in the KPC transcriptome compared to the WT transcriptome. We found that more than 400 genes were direct targets of FOSL2 (Fig. 6b). These targets were enriched for GO terms, such as epithelial cell proliferation, DNA-binding, and leucocyte migration (Fig. 6c). Among these candidate targets, we observed that the CCL28 gene had more FOSL2 occupancy in its upstream region (Fig. 6d) than other targets, such as Capa3, Cdk1, and Cdk4 (Fig. S5D). Previous reports have shown that CCL28 is not only implicated in mucosal immunity but also involved in tumour immunosuppression [44–46]. Our results also showed that FOSL2 could regulate immune cell infiltration in PDAC (Fig. 5i–k). These findings implied that CCL28 mediated FOSL2’s role in shaping the tumour immune microenvironment. Therefore, we explored whether CCL28 was a genuine target of FOSL2. IHC showed that CCL28 was up-regulated in KC and KPC mice compared to WT mice (Fig. 6e). TCGA bulk RNA-seq also showed higher CCL28 expression in PDAC tumour tissues than in normal tissues (Fig. S5E) and was positively correlated with FOSL2 (Fig. S5F). Similar to FOSL2, the high CCL28 group was associated with a poor outcome in patients with PDAC (Fig. 6f). It has been reported that CCL28 is mainly expressed in epithelial cells of various mucosal tissues [47]. In line with this, we also observed that CCL28 was mainly expressed in pancreatic ductal cells rather than other cell types (Fig. 6g). We found higher CCL28 expression in the high MUC5AC group than in the low MUC5AC group (Fig. 6h), indicating that the expression of CCL28 was up-regulated in cancerous ductal cells compared with normal pancreatic ductal cells. scRNA-seq also showed higher CCL28 expression in the high FOSL2 group than the low FOSL2 group in PDAC ductal cells (Fig. 6i).

In our present study, we observed that knockdown of FOSL2 using two independent shRNAs (Fig. S5G) led to a decrease in the expression of CCL28 at the mRNA and protein levels in MIA PaCa-2 and PANC-1 cells, as measured by RT-qPCR and ELISA, respectively (Fig. 6j, l). Conversely, the overexpression of FOSL2 (Fig. S5G) resulted in the up-regulation of CCL28 in PanO2 cells (Fig. 6k, m). We further investigated the molecular mechanism by which FOSL2 up-regulated CCL28. We searched a 12-kb human CCL28 upstream region of the transcription start site (TSS) and selected four putative FOSL2 binding sites (−1709 bp, −4192 bp, −8200 bp, and −11,414 bp) before designing and synthesising the corresponding primers. FOSL2 ChIP-qPCR analysis in MIA PaCa-2 and PANC-1 cells showed that only binding site 3 (BS3) was enriched in FOSL2 (Fig. 6n, o).

Subsequently, we constructed wild-type (WT) and mutant-type (Mut) CCL28 luciferase reporter plasmids in the −8 to −12 kb region of CCL28 and performed DLR gene assays (Fig. 6p). The results showed that FOSL2 knockdown significantly reduced CCL28 DLR activity after the co-transfection of wild-type CCL28. However, luciferase activity remained unchanged when co-transfected with the mutant CCL28 plasmid in MIA PaCa-2 and PANC-1 cells (Fig. 6q, r). We also confirmed these results in 293 T cells (Fig. S5H, I). Taken together, these findings demonstrated that CCL28 was a direct transcriptional target of FOSL2, and its transcriptional activation occurred through FOSL2 binding to BS3.

### FOSL2 promotes tumour growth and Treg cell recruitment through CCL28

**Fig. 7:**
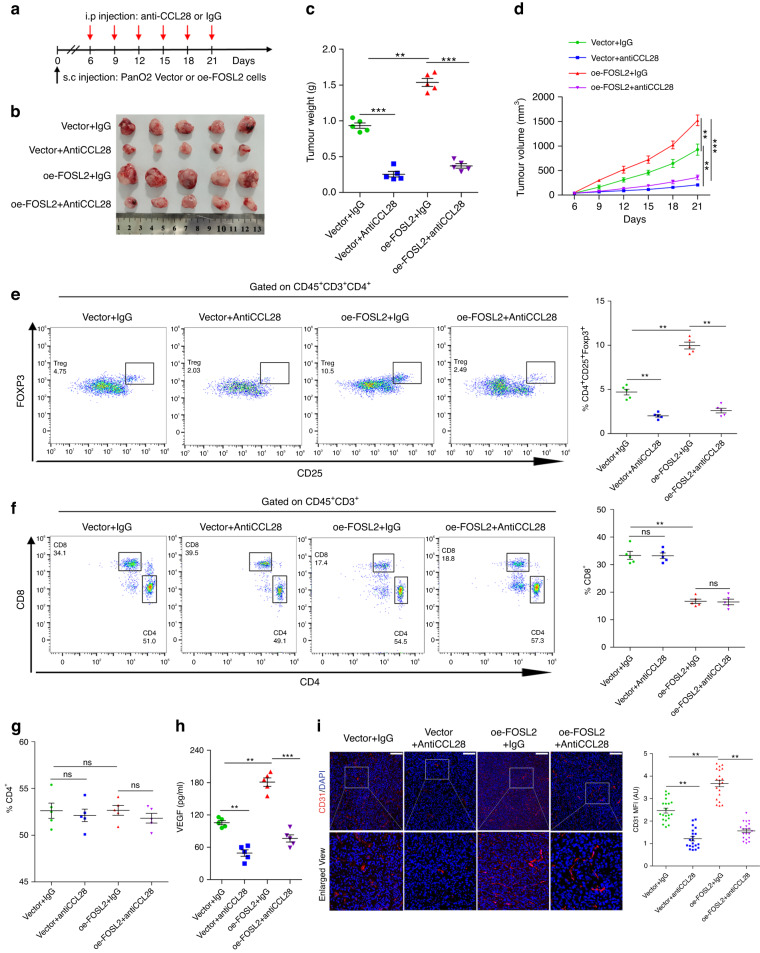
FOSL2 promotes Treg cell recruitment through CCL28. **a**–**d** Scheme representing the experimental procedure (**a**), representative tumour image (**b**), tumour weight (**c**), and tumour growth curves (**d**) of C57BL/6 mice injected subcutaneously with Vector and oe-FOSL2 PanO2 cells with treatment of CCL28 neutralising antibody or IgG. **e** Representative flow staining (left panel) and frequency (right panel) of Treg cells in tumour xenograft of indicated groups. **f** Representative flow staining (left panel) and frequency (right panel) of CD8^+^ T cells in tumour xenograft of indicated groups. **g** The frequency of CD4^+^ T cells in tumour xenograft of indicated groups. **h** The level of VEGFA protein in the serum of Vector or oe-FOSL2 mice following administration of anti-CCL28 antibody or IgG, as determined by ELISA. **i** Representative immunofluorescence staining (left panel) and quantitation (right panel) of mean fluorescence intensity of CD31 in indicated groups. Data are representative shown as mean ± SD or SEM (**P < 0.01; ***P < 0.001; ns not significant).

It has been reported that CCL28 can recruit Treg cells, causing tumour immunosuppression [44–46]. Our results also showed that FOSL2 could recruit Treg cells in vivo (Fig. 5j). Therefore, we wanted to explore whether FOSL2 recruited Treg cells through CCL28 in PDAC. C57BL/6 mice subcutaneously transplanted with vector or FOSL2-overexpressed PanO2 cells were given anti-CCL28 monoclonal antibody or isotype IgG by intraperitoneal injection (Fig. 7a). Interestingly, anti-CCL28 treatment significantly reduced tumour size compared to the isotype control (Fig. 7b–d). The ratio of Treg cells was significantly decreased in tumour tissues of anti-CCL28-treated mice (Fig. 7e), but the ratios of CD8^+^ and CD4^+^ T cells were not changed by anti-CCL28 treatment in tumour tissues (Fig. 7f, g).

Previous studies have reported an inverse correlation between angiogenesis and tumour-infiltrating T cells [48, 49]. FOSL2 expression is reported to be positively correlated with angiogenesis in breast cancer [50]. We found a significantly increased amount of vascular endothelial growth factor A (VEGFA) in the mouse serum with FOSL2 overexpression than that with control but was rescued with anti-CCL28 treatment upon FOSL2 overexpression (Fig. 7h). Immunofluorescence staining for CD31 indicated increased angiogenesis in oe-FOSL2 tumour but was reversed with anti-CCL28-treated tumours (Fig. 7i). In conclusion, these results suggested that CCL28 mediated FOSL2-driven tumour growth and Treg cell infiltration.

### Mutant KRAS regulates FOSL2 expression through MAPK/ERK signalling pathway

**Fig. 8:**
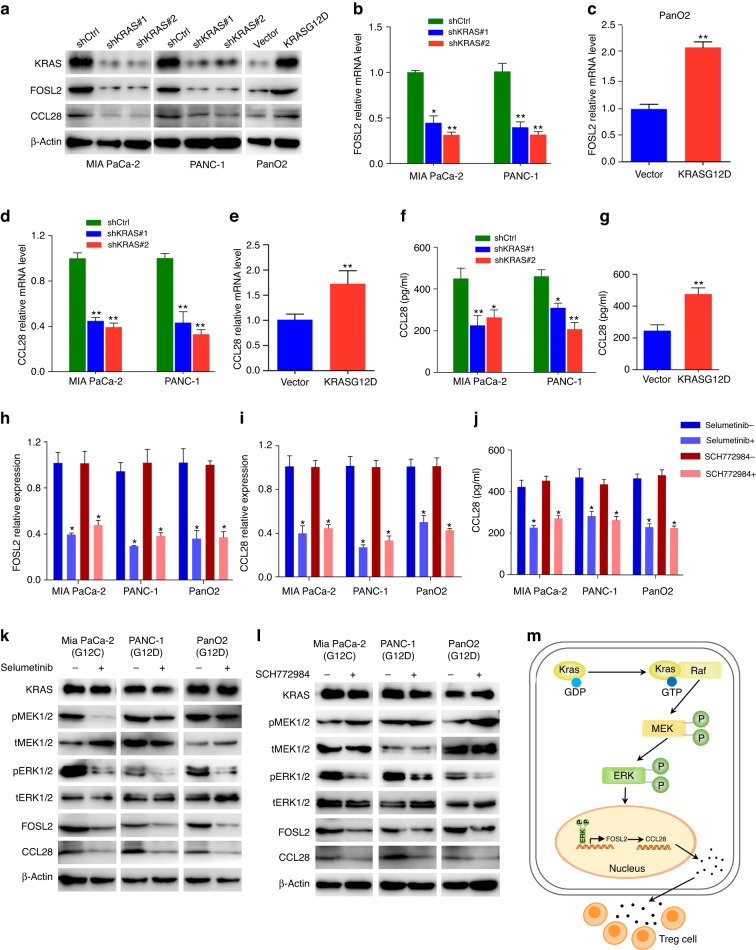
KRAS up-regulates FOSL2 expression through MAPK/ERK pathway. **a** FOSL2 and CCL28 protein levels in MIA PaCa-2 and PANC-1 cells with KRAS knockdown or in PanO2 cells with mutant KRAS overexpression. **b**, **c** FOSL2 mRNA levels in MIA PaCa-2 and PANC-1 cells with KRAS knockdown (**b**) or in PanO2 cells with mutant KRAS overexpression (**c**). For (**b**), the shKRAS#1 and shKRAS#2 groups were compared with the shCtrl group, respectively. **d**, **e** CCL28 mRNA levels in MIA PaCa-2 and PANC-1 cells with KRAS knockdown (**d**) or in PanO2 cells with mutant KRAS overexpression (**e**). For (**d**), the shKRAS#1 and shKRAS#2 groups were compared with the shCtrl group, respectively. **f**, **g** CCL28 protein in supernatants from MIA PaCa-2 and PANC-1 cells with KRAS knockdown (**f**) or from PanO2 cells with mutant KRAS overexpression (**g**), as determined by ELISA. For (**f**), the shKRAS#1 and shKRAS#2 groups were compared with the shCtrl group, respectively. **h**, **i** RT-qPCR showed the FOSL2 (**h**) and CCL28 (**i**) mRNA levels in MIA PaCa-2(G12C), PANC-1(G12D), and PanO2(G12D) cells with or without KRAS/MAPK inhibitors (Selumetinib and SCH772984). **j** CCL28 protein in supernatants from MIA PaCa-2(G12C), PANC-1(G12D), and PanO2(G12D) cells with or without KRAS/MAPK inhibitors (Selumetinib and SCH772984), as determined by ELISA. **k**, **l** Western blotting showed the KRAS, pMEK1/2, tMEK1/2, pERK1/2, tERK1/2, FOSL2, and CCL28 protein levels from MIA PaCa-2(G12C), PANC-1(G12D), and PanO2(G12D) cells with or without KRAS/MAPK inhibitors Selumetinib (**k**) and SCH772984 (**l**). **m** The mechanistic illustration showed KRAS/MAPK-FOSL2-CCL28-Treg signalling axis in PDAC progression. FOSL2 binds to the proximity of the chemokine gene CCL28 and promotes its transcription and secretion. CCL28 recruits regulatory T cells and leads to tumour immune evasion. Data are representative of at least three independent experiments and shown as mean ± SEM. *P < 0.05; **P < 0.01.

We showed that FOSL2 expression was higher in KPC and KC mice than in WT mice, although the difference was not significant between the KPC and KC groups (Fig. 3g–i), suggesting that KRAS mutation was involved in the regulation of FOSL2. From scRNA-seq data, we observed that KRAS expression was higher in endothelial and ductal cells than in other types of cells (Fig. S6A). To further investigate the relationship between KRAS and FOSL2, we performed KRAS knockdown in MIAPaCa-2 (KRAS^G12C^) and PANC-1 (KRAS^G12D^) cells. Our results showed that KRAS depletion resulted in the down-regulation of FOSL2 at the mRNA and protein levels, as demonstrated by RT-qPCR and Western blotting analysis (Fig. 8a, b). Conversely, overexpression of KRAS^G12D^ in the PanO2 cells increased the mRNA and protein levels of FOSL2 (Fig. 8a, c). It is worth noting that KRAS mutations are involved in up to 96% of PDACs, 52% of colorectal carcinomas, and 32% of lung adenocarcinomas [51]. Based on TCGA data, there was a positive correlation between FOSL2 and KRAS expression in PDAC, colorectal carcinomas, and lung adenocarcinomas (Fig. S6B–D). Furthermore, scRNA-seq showed that FOSL2 was positively correlated with KRAS in PDAC ductal cells (Fig. S6E). We also found that the depletion of KRAS decreased the expression of CCL28 at the mRNA and protein levels in the MIA PaCa-2 and PANC-1 cells, as confirmed by Western blotting analysis, RT-qPCR, and ELISA (Fig. 8a, d, f). In contrast, the ectopic expression of KRAS^G12D^ led to the up-regulation of CCL28 in PanO2 cells (Fig. 8a, e, g). Based on TCGA data, there was a positive correlation between CCL28 and KRAS expressions in PDAC and colorectal carcinomas (Fig. S6F, G). These findings suggested that KRAS regulated the expression of FOSL2 and its target CCL28.

RAS activation is known to drive RAF/MEK/ERK cascades, which are part of the ERK signalling pathway. To understand the mechanism by which KRAS up-regulated FOSL2, we used specific inhibitors of MEK (Selumetinib) and ERK (SCH772984). We observed that the expression of FOSL2, both in endogenous KRAS mutation cells (MIAPaCa-2 and PANC-1) and in exogenously transfected PanO2 cells with KRAS^G12D^, was reduced upon treatment with the MEK inhibitor Selumetinib, as seen by RT-qPCR and Western blotting analysis (Fig. 8h, k). We also observed a similar effect upon partial inhibition of ERK with SCH772984 (Fig. 8h, l). Furthermore, we found that both inhibitors reduced the expression of CCL28, as measured by RT-qPCR, ELISA, and Western blotting analysis (Fig. 8i–l). These findings suggested that mutant KRAS mediated FOSL2 transcription and subsequent CCL28 expression via the MAPK/ERK pathway.

## Discussion

In this study, we conducted a comprehensive evaluation of epigenomic changes in a well-established GEMM of PDAC. The model included mice with wild-type (WT), Kras^G12D^ mutant (KC), and Kras^G12D^/P53^R172H^ double mutant (KPC) genotypes, with or without KRAS and/or P53 mutations. We integrated these data with RNA-seq data from the same mice and identified approximately 35,000 differentially accessible regions in at least one pairwise comparison. Clustering and motif analysis of these regions revealed cluster-specific TFs that played important roles in PDAC progression.

Furthermore, we provided the first evidence that mutant KRAS up-regulated FOSL2 expression through ERK signalling. Up-regulated FOSL2 transcriptionally activated CCL28 expression by binding to its upstream 8100–8200 bp regions, which in turn recruited more Treg cells and shaped an immunosuppressive microenvironment in PDAC. Importantly, our study demonstrated that CCL28 blockade upon FOSL2 overexpression exerted a potent anti-tumour effect by suppressing Treg cell infiltration and might serve as a potential therapeutic target for pancreatic cancer.

FOSL2 belongs to the FOS family of TFs and forms the AP1 complexes by binding to JUN family TFs. The AP1 complexes play a central role in the transcriptional regulation of almost all areas of eukaryotic cellular behaviour, including stress response, proliferation, and development [52]. The oncogenic activity of FOSL2 has been reported in breast cancer [50], ovarian cancer [53], colon cancer [54], and hepatocellular carcinoma [55]. In our present study, we also observed an oncogenic role of FOSL2 through both immune and non-immune mechanisms. In the non-immune pathway, we found that FOSL2 promoted PDAC cell proliferation, migration, and invasion in vitro. However, the precise mechanism underlying this requires further investigation. Studies have reported that FOSL2 can interact with Smad3, Wnt5a, or SNAI2 to promote tumour growth [50, 56, 57]. Thus, further studies are needed to determine whether FOSL2 promotes PDAC progression through these mechanisms. In the immune pathway, we found that FOSL2 promoted tumour growth, decreased CD8^+^ T cell infiltration, and increased Treg cell recruitment in immunocompetent C57BL/6 mice. These results suggested that FOSL2 participated in tumour progression through different regulatory mechanisms. Hence, exploring the functions and mechanisms of FOSL2 in PDAC and other cancers holds significant value.

It is widely recognised that CCL28, a member of the CC (β-chemokine) family, plays a crucial role in regulating immune cell migration and modulating the immune microenvironment [58]. The up-regulation of CCL28 in tumour cells has been shown to facilitate Treg cell infiltration into tumour tissues, thus creating an immunosuppressive microenvironment that favours tumourigenesis [45]. In the present study, we identified CCL28 as a key mediator linking FOSL2 and the pancreatic tumour microenvironment. Mechanistically, we found that FOSL2 bound to the upstream region of CCL28 to activate its expression, leading to the recruitment of Treg cells and highlighting the importance of the FOSL2-CCL28-Treg axis in pancreatic cancer. Functionally, we observed that FOSL2 overexpression increased tumour growth and Treg cell infiltration, which could be reversed by blockade of CCL28 activity in vivo, indicating its potential as a therapeutic target. Although there are currently no inhibitors targeting FOSL2 for cancer treatment, targeting downstream effectors of FOSL2 may provide an alternative strategy. Notably, neutralising CCL28 with an antibody decreases Treg cell infiltration and suppresses tumour growth, as has been shown in gastric and ovarian cancer [45, 46]. Taken together, our findings suggested that CCL28 might represent a promising therapeutic target for pancreatic cancer.

Furthermore, a recent study has suggested that CCL28 deficiency significantly attenuates the growth of subcutaneous tumours in both immunocompetent syngeneic mice and immunodeficient NOD-SCID mice [59]. Our results also showed that FOSL2 depletion markedly reduced tumour cell proliferation. These findings suggested that FOSL2 and CCL28 might also regulate tumour growth in an immune-independent manner, potentially through cell autonomous mechanisms that require further investigation.

The RAS/MAPK pathway plays a central role in human cancer as it is hyperactivated in a wide range of tumours, and many of its elements have been identified as oncogenes. KRAS mutations are implicated in up to 96% of PDAC cases and are considered an initiating event in PDAC oncogenesis [8, 9]. KRAS activates the MAPK cascade, which culminates in the stimulation of kinase activity toward the MEK1/2 dual-specificity kinases, which in turn phosphorylate and activate the ERK family of kinases. ERK kinases translocate to the nucleus and phosphorylate a broad spectrum of substrates involved in various processes, such as proliferation, survival, and differentiation [60]. It has been reported that the ERK1/2-FOSL2 pathway is involved in the regulation of EMT and metastasis of non-small-cell lung carcinoma (NSCLC) [56]. We found that the regulatory effect of KRAS on FOSL2 was mediated by the MAPK/ERK pathway. The expressions of FOSL2 and CCL28 were inhibited in the presence of inhibitors of MEK or ERK. Further investigation is needed to determine if the positive regulation of CCL28 by KRAS is mediated by FOSL2.

It is well known that AP-1 factors typically function by forming dimers with other members of the family [61, 62], suggesting that other AP-1 members may interact with FOSL2 as well. In our present study, we did not investigate whether FOSL2 could interact with other AP-1 members, which warrants further investigation. Furthermore, we did not examine whether other AP-1 members showed expression correlation with CCL28 and KRAS, or whether they regulated tumour growth. However, previous reports have shown that FOS transcription can mediate YAP/TAZ to promote cell proliferation [63], JUNB can promote cell cycle progression by inducing cyclin E1 and repressing transforming growth factor (TGF)-β2 genes [64], and CRTC1 can associate with c-Jun and c-Fos to activate transcription and promote cellular proliferation [65]. To date, no studies have explored the association between other AP-1 families and CCL28. Therefore, we, for the first time, found that FOSL2 could regulate the expression of CCL28. Future studies should investigate the association between other AP-1 factors and CCL28. Additionally, a previous report has demonstrated that mutant KRAS can promote FOSL1 expression [66]. Consistent with this finding, we also found that KRAS could regulate the transcription of FOSL2.

In summary, our study shed light on the evolving epigenetic changes in PDAC and found that the TF FOSL2 was a downstream target of mutant KRAS, which promoted immunosuppression by increasing the expression of CCL28. This chemokine, in turn, recruited Treg cells, contributing to the immune evasion of PDAC. Our findings suggested that the KRAS–FOSL2–CCL28–Treg cell axis played a regulatory role in PDAC immunosuppression. We proposed that targeting CCL28 to prevent Treg cell recruitment could be a highly effective and selective strategy to overcome immune evasion in PDAC patients.

## Supplementary information

Supplementary Figure legend

Figure S1

Figure S2

Figure S3

Figure S4

Figure S5

Figure S6

Table S1

Table S2

Table S3

Table S4

Table S5

## Data Availability

The sequencing data were uploaded to GEO. The accession numbers for the bulk ATAC-seq, H3K27ac ChIP-seq, RNA-seq, and FOSL2 CUT&Tag reported in this paper are GSE190900, GSE190901, GSE190903, and GSE190912, respectively.
