# Supplementary material for: Chromatin accessibility uncovers KRAS-driven FOSL2 promoting pancreatic ductal adenocarcinoma progression through up-regulation of CCL28
Source: Br J Cancer. 2023 Jun 28;129(3):426–43. doi: 10.1038/s41416-023-02313-y (PMC10403592; doi:10.1038/s41416-023-02313-y)
Supplement: Supplementary file 1 — Supplementary Figure legend [file 41416_2023_2313_MOESM1_ESM.docx]

**­­­­ Figure S1**

**Global epigenomic profile in PDAC mouse model**

A. Representative H&E-stained sections for 6-month-old WT pancreas, KC, and KPC pancreatic cancer tissues. Scale bar, 100 µm.

B. IHC images of representative WT pancreas, KC, and KPC neoplastic tissues stained for Ki-67. Representative images (left) and quantification (right). Scale bar, 100 µm. ­­­­**P < 0.01; **P < 0.001.

C-E. PCA of the ATAC-seq (C), ChIP-seq (D), and RNA-seq (E) profiles for WT, KC, and KPC mice. The plot shows the percentage of variance explained by the Dim1 and Dim2 axes and the distribution of the samples along the two first principal components.

F. Pie plots showed the genomic annotation of all accessible regions.

G-I. Scatterplots of mean ATAC-seq counts per peak comparing the indicated samples. Red indicates differential peaks enriched in KPC compared with WT or KC; Blue indicates differential peaks enriched in KC compared with WT or KPC; Green indicates differential peaks enriched in WT compared with KC or KPC; Black indicates common peaks whose fold-change is less than 3; Grey indicates the rest of peaks whose fold-change is greater than 3 and adjusted p-value greater than 1x10^-3^. The upper-right number showed the count of common peaks.

J-L. Scatterplots of mean RNA-seq transcriptome comparing the indicated samples (left), with points for differentially expressed genes colored, from two independent replicates for each group. Boxplots of RNA-seq counts per transcript from the indicated samples (bottom labels) for DEGs in the comparison labeled at the top (right). For boxplots in J-L, boxes indicate an interquartile range with whiskers +/-1.5 times this range and outlier points from 2 independent replicates for each group. *P < 0.05; **P < 0.01; ns, not significant.

**Figure S2**

**The gene associations of differentially accessible regions in WT and KC, and KPC mice**

A and B. BETA for the association between gain or loss peaks of ATAC-seq and gene expression from the indicated groups (labeled above). The red line indicates up-regulated genes, the purple line indicates downregulated genes, the dashed line indicates the non-differential genes as background.

C and D. Venn diagrams illustrate the intersection of differentially accessible regions from pairwise comparisons of WT, KC, and KPC mice characterizing regions "specific" to a subset (C) or "not" in a subset (D). "Specific" indicates peaks enriched in the indicated group (labeled at the bottom) but absent in the other two groups. "Not" indicates peaks absent in the indicated group (labeled at the bottom) but enriched in the other two groups.

E and F. Venn diagrams illustrate the intersection of DEGs from pairwise comparisons of WT, KC, and KPC mice characterizing regions "specific" to a subset (E) or "not" in a subset (F). "Specific" indicates up-regulated genes in the indicated group (labeled at the bottom) but down-regulated in the other two groups. "Not" indicates down-regulated genes in the indicated group (labeled at the bottom) but up-regulated in the other two groups.

G. Dotplot showing the representative gene ontology and pathway terms of "specific" and "not" accessible regions-annotated genes in WT, KC, and KPC mice. Representative pathways are presented by the dotplot function of the R package clusterProfiler based on the adjusted p-value. The color key from blue to red indicates the adjusted p-value.

H. Dotplot showing the representative gene ontology and pathway terms of "specific" and "not" DEGs in WT, KC, and KPC mice. The color key from blue to red indicates the adjusted p-value. Note these representative pathways are not enriched in KC "specific", so the KC group is not shown in "specific".

I. Boxplots of RNA-seq counts per transcript from the indicated samples (bottom labels) for peak-annotated genes in clusters 1-5. Boxes indicate an interquartile range with whiskers +/-1.5 times this range and outlier points from 2 independent replicates for each group. *P < 0.05; **P < 0.01. n.s. not significant.

J. Pie plot showed the genomic annotation of all H3K27ac signals.

**Figure S3**

**Representative markers for main cell types in PDAC**

1. The t-distributed stochastic neighbor embedding (t-SNE) plot demonstrates the main clusters in PDAC.
2. Violin plots displaying the expression of well-known representative markers across the cell types identified in PDAC. The y-axis shows the normalized read count.
3. The expression level of MUC5AC for main cell types is plotted onto the t-SNE map. The color key from gray to purple indicates relative expression levels from low to high. The "expression level" was normalized by the logNormalize method in Seurat.
4. Violin plot showing the global MUC5AC expression level from scRNA-seq in the low MUC5AC ductal cell group and high MUC5AC ductal cell group.

**Figure S4**

**FOSL2 promotes PDAC progression *in vitro* and *in vivo***

A and B. RT-qPCR analysis of FOSL2 expression in MIA PaCa-2 and PANC-1 cells with FOSL2 knockdown (A), or in MIA PaCa-2, PANC-1, and PanO2 cells with FOSL2 overexpression (B).

C. FOSL2 protein levels in MIA PaCa-2 and PANC-1 cells with FOSL2 knockdown or in MIA PaCa-2, PANC-1, and PanO2 cells with FOSL2 overexpression.

D and E. Proliferation evaluation in MIA PaCa-2 (D) and PANC-1 cells (E) with FOSL2 knockdown by CCK-8 assay. n = 3 for each time point.

F-I. Representative micrographs (left and middle) and quantification (right) of the transwell assay show the migration and invasiveness in MIA PaCa-2 (F and H) and PANC-1 cells (G and I) with FOSL2 knockdown.

J-L. Proliferation evaluation in MIA PaCa-2 (J), PANC-1 (K), and PanO2 cells (L) with FOSL2 overexpression by CCK8 assay. n = 3 for each time point.

M-O. Representative micrographs (top) and quantification (bottom) of the transwell assay show the migration and invasiveness in MIA PaCa-2 (M), PANC-1 (N), and PanO2 cells (O) with FOSL2 overexpression.

Data are representative of at least three independent experiments and shown as mean ± SEM (*p < 0.05; **p < 0.01).

**Figure S5**

**CCL28 is a direct target gene of FOSL2**

A. Average read density of FOSL2 CUT&Tag in KPC mice.

B. Scatter plot showing the agreement between biological replicate FOSL2 CUT&Tag in KPC mice. Each dot is a peak in the CUT&Tag data. R=Pearson's R correlation coefficient.

C. Co-TF motif analysis shows enrichment motifs for FOSL2 CUT&Tag in KPC mice.

D. CUT&Tag signal track showing Casp3, Cdk1, and Cdk4 associated FOSL2 occupancy at promoter regions in KPC mice.

E. Boxplots show the expression level of CCL28 in PDAC patients from TCGA data. **P < 0.01.

F. Correlation of CCL28 and FOSL2 mRNA expressions in PDAC patients from TCGA data.

G. Immunoblot shows the abundance of FOSL2 in MIA PaCa-2, PANC-1, and PanO2 cells before and after knockdown or overexpression.

H. Immunoblot shows the abundance of FOSL2 in 293T cells before and after knockdown. siFOSL2: small interfering FOSL2.

I. Luciferase reporter activities of CCL28 upstream or upstream with mutated binding sites in 293T cells with FOSL2 knockdown. The siFOSL2#1 and siFOSL2#2 groups were compared with the siCtrl group, respectively.

**Figure S6**

**The correlation between FOSL2, CCL28, and KRAS in malignant cancer samples**

A. Expression level of KRAS for main cell types are plotted onto the t-SNE map. The color key from gray to purple indicates relative expression levels from low to high. The "expression level" was normalized by the logNormalize method in Seurat.

B-D. The correlation between the expression of FOSL2 and KRAS in mRNA levels in malignant cancer patients (labeled above) from TCGA data.

E. Expression levels of KRAS and FOSL2 genes in ductal cells are plotted onto the t-SNE map. The color key from gray to purple indicates relative expression levels from low to high.

F and G. The correlation between the expression of CCL28 and KRAS in mRNA levels in malignant cancer patients (labeled above) from TCGA data.
