# Supplementary figures and images for: Chromatin accessibility uncovers KRAS-driven FOSL2 promoting pancreatic ductal adenocarcinoma progression through up-regulation of CCL28
Source: Br J Cancer. 2023 Jun 28;129(3):426–43. doi: 10.1038/s41416-023-02313-y (PMC10403592; doi:10.1038/s41416-023-02313-y)

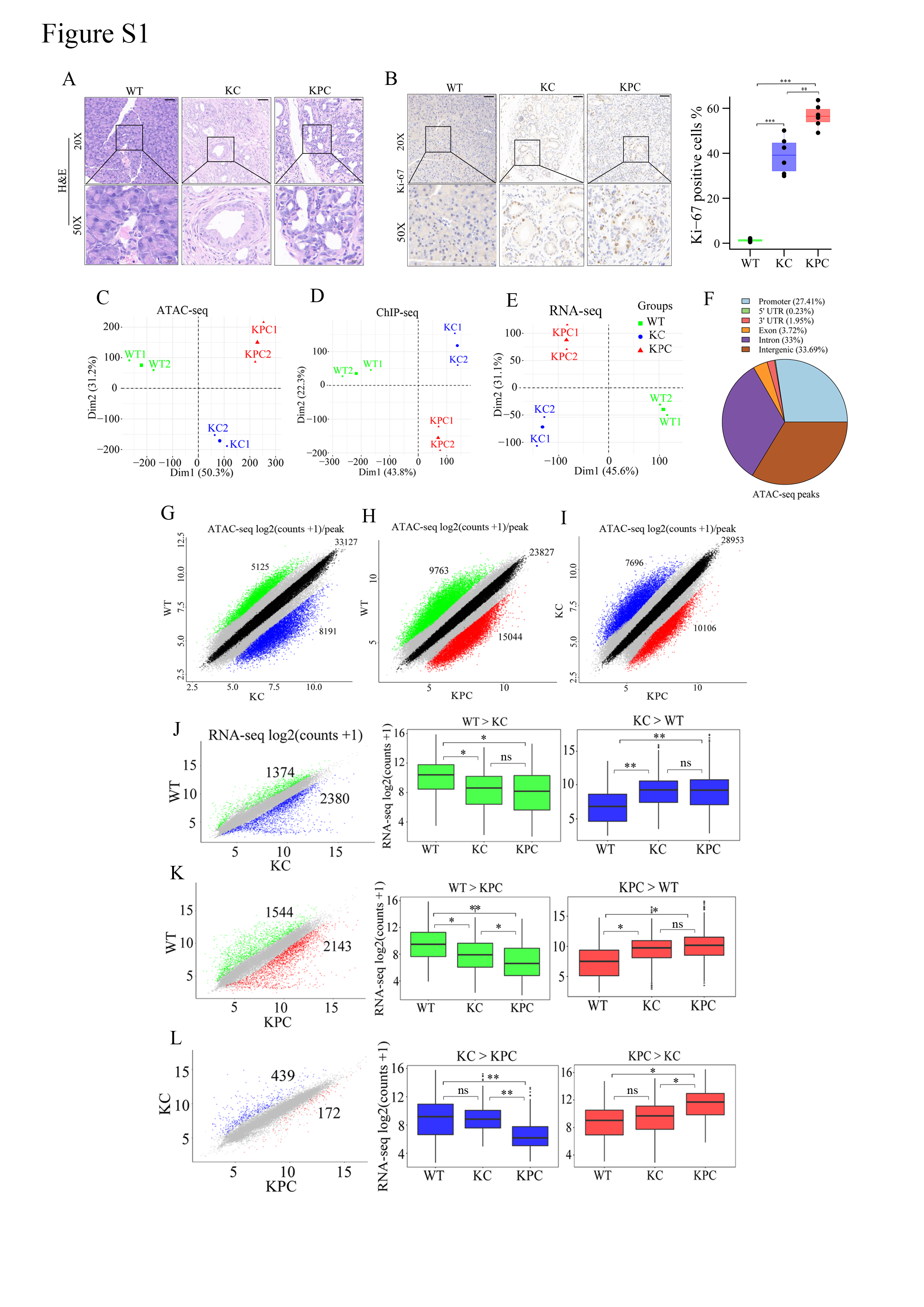

Supplement: Supplementary file 2 — Figure S1 [file 41416_2023_2313_MOESM2_ESM.tif]

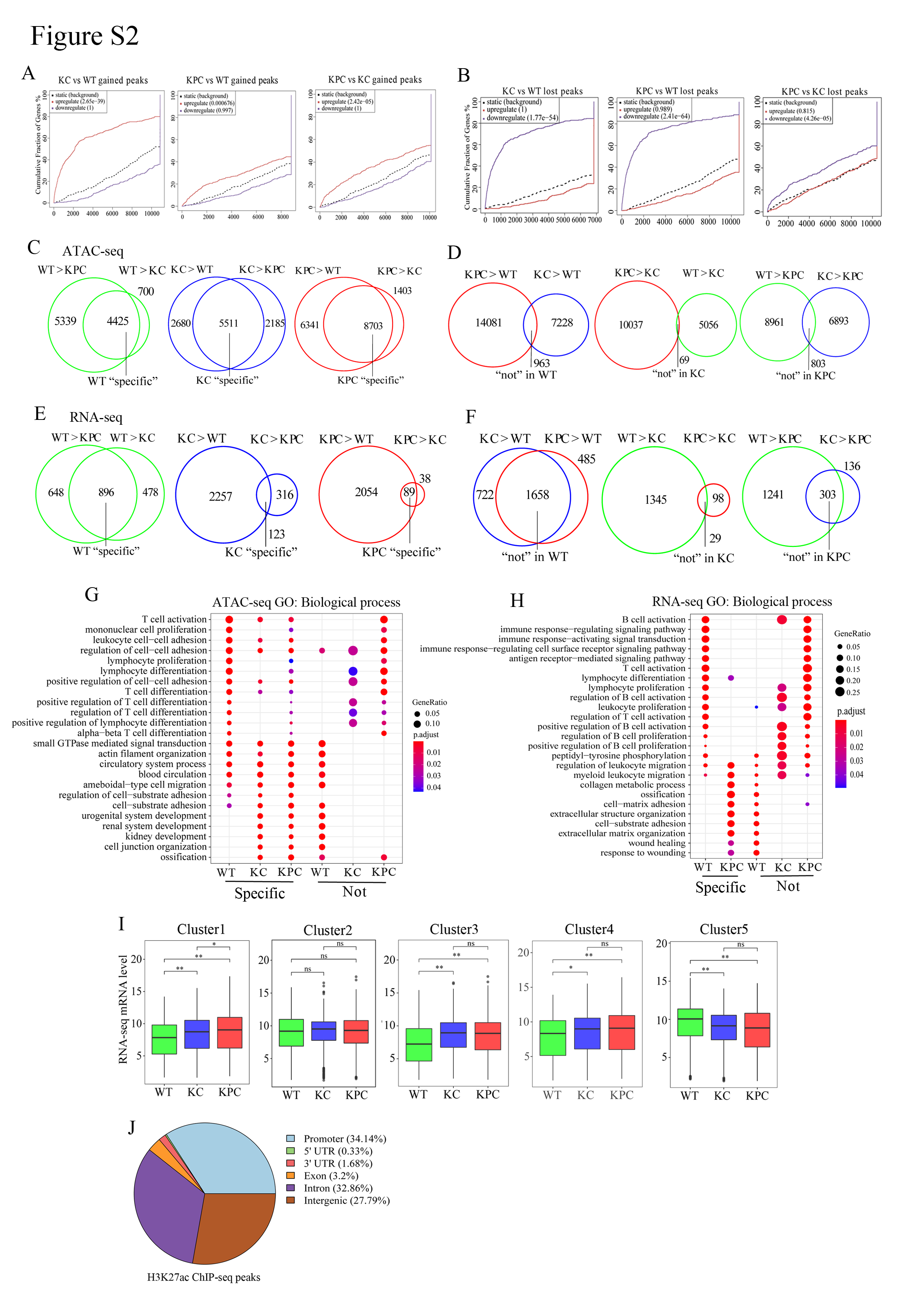

Supplement: Supplementary file 3 — Figure S2 [file 41416_2023_2313_MOESM3_ESM.tif]

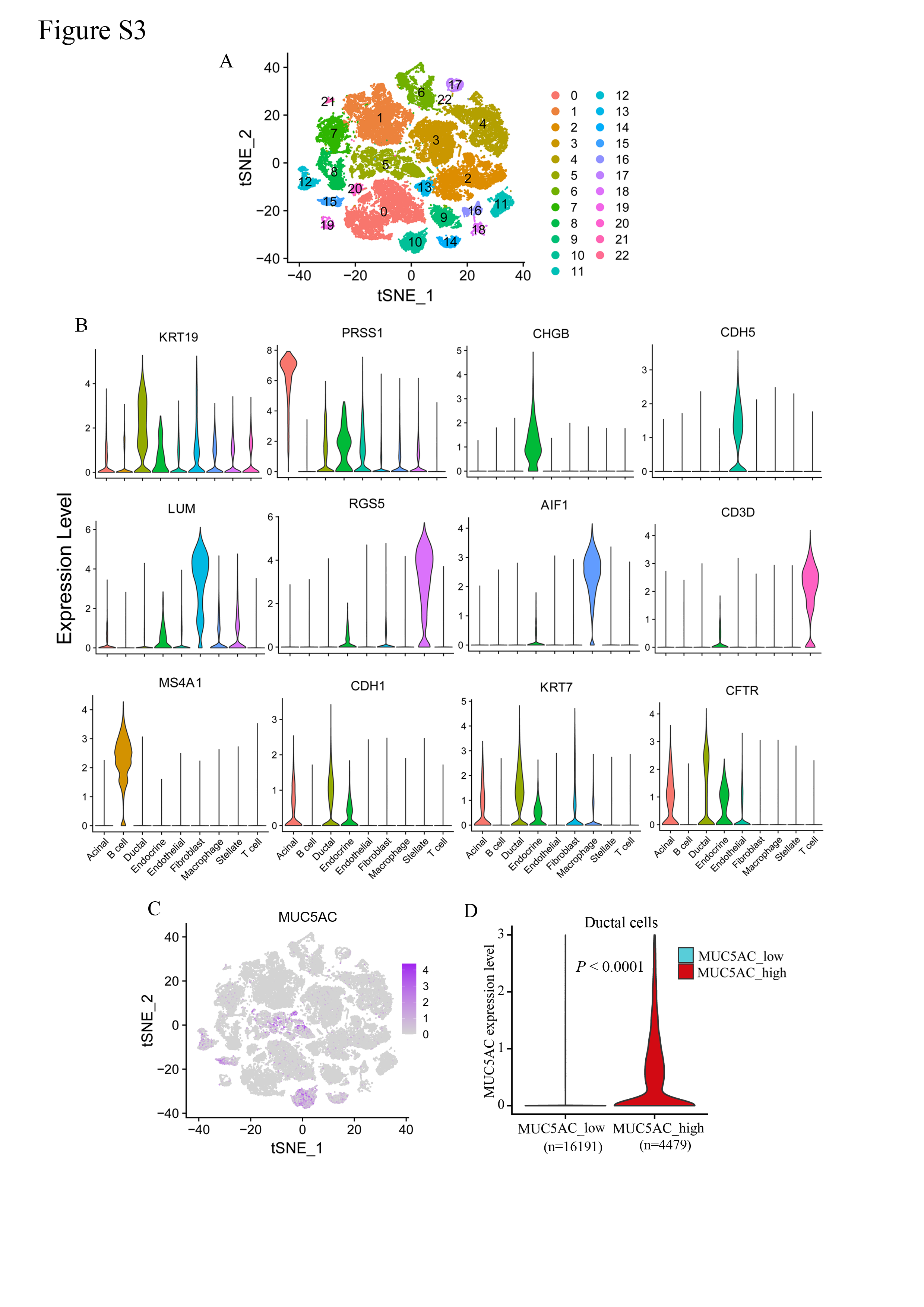

Supplement: Supplementary file 4 — Figure S3 [file 41416_2023_2313_MOESM4_ESM.tif]

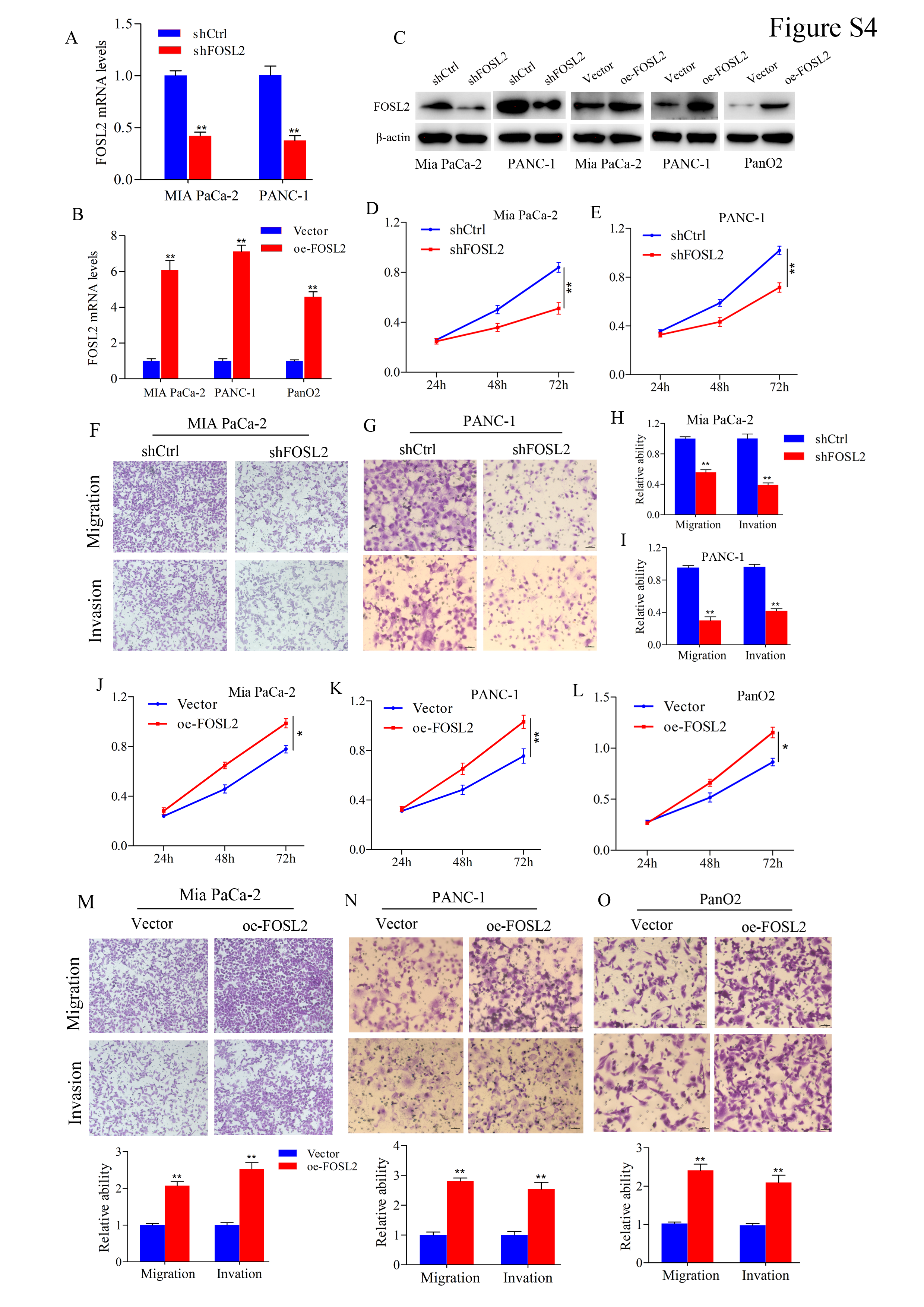

Supplement: Supplementary file 5 — Figure S4 [file 41416_2023_2313_MOESM5_ESM.tif]

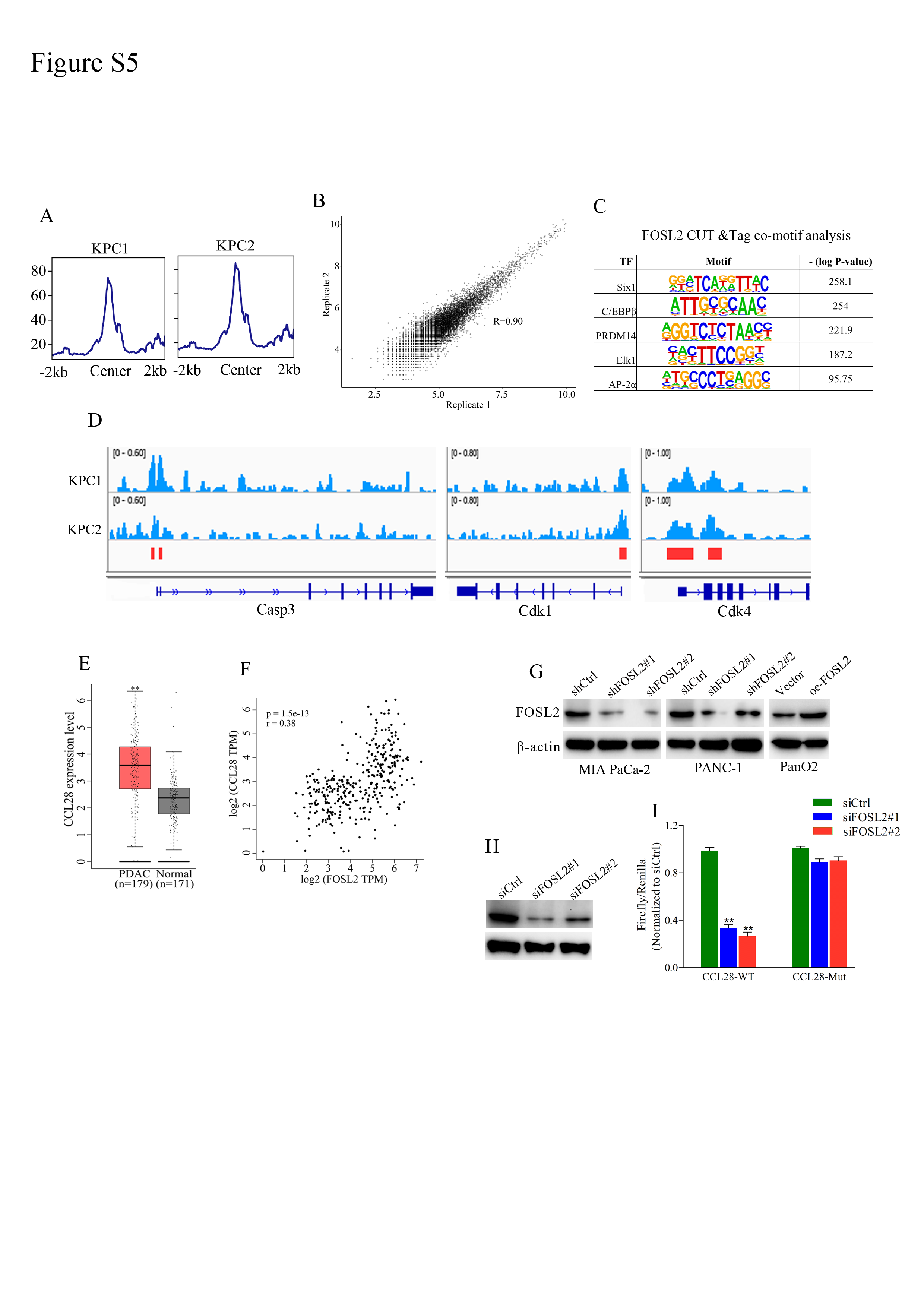

Supplement: Supplementary file 6 — Figure S5 [file 41416_2023_2313_MOESM6_ESM.tif]

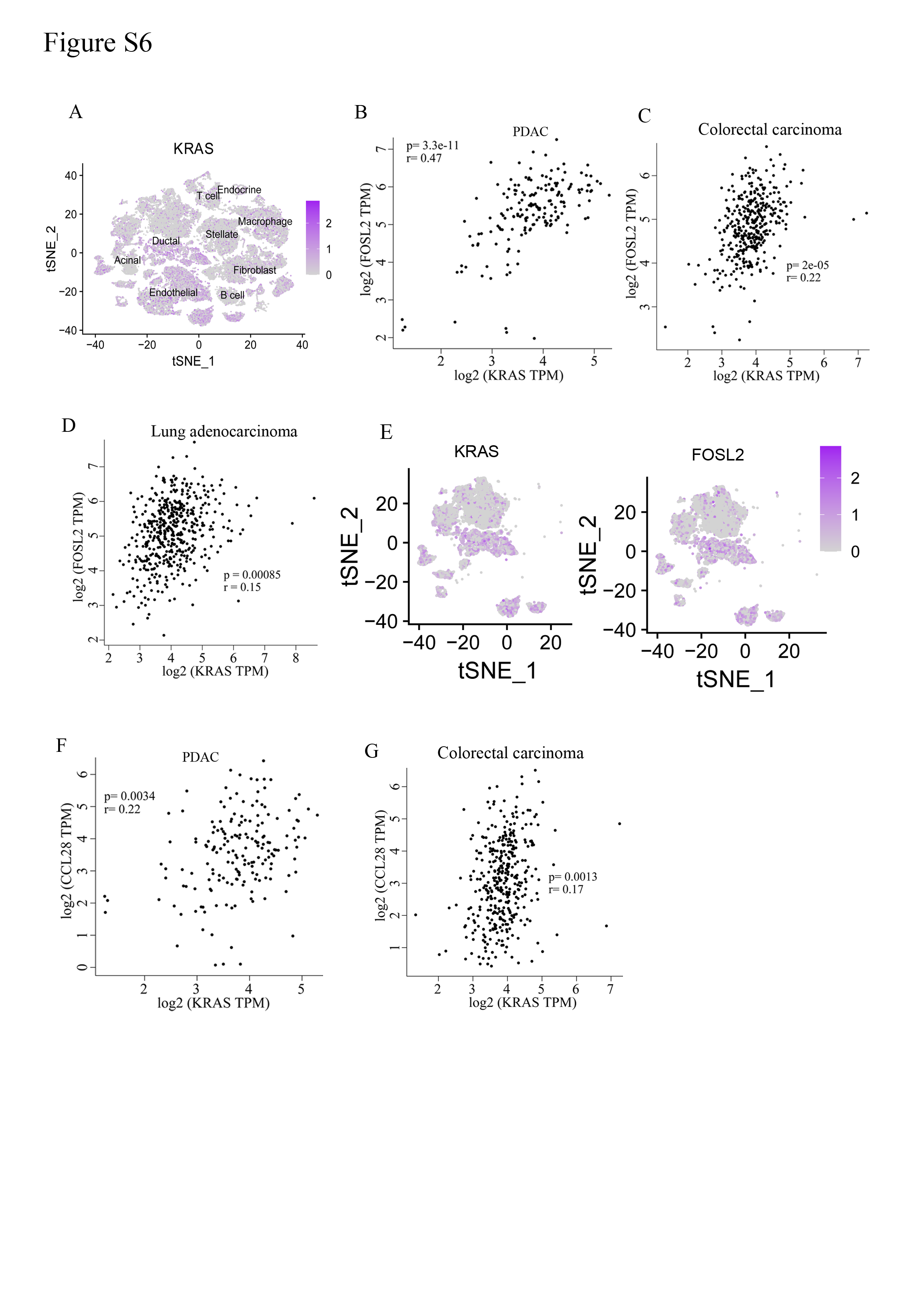

Supplement: Supplementary file 7 — Figure S6 [file 41416_2023_2313_MOESM7_ESM.tif]
